# Maternal acute SARS-CoV-2 infection impairs preimplantation embryo development and reprograms the early offspring hematopoietic system

**DOI:** 10.1038/s41421-025-00856-3

**Published:** 2025-12-23

**Authors:** Meiling Zhang, Di Liu, Songmao Li, Jiansheng Liu, Fanghao Guo, Haibin Zhu, Li Zhang, Di Sun, Yu Yan, Yanquan Li, Rui Qiao, Haixia Ding, Qing Zhang, Mengxi Guo, Yongjian Ma, Zhiwei Liu, Wen Li, Yuxuan Zheng

**Affiliations:** 1 International Peace Maternity and Child Health Hospital, Shanghai Key Laboratory of Embryo Original Disease, Shanghai Jiao Tong University School of Medicine, Shanghai, China; 2 Human Phenome Institute, Pudong Hospital, Fudan University, Shanghai, China

**Keywords:** Reprogramming, Epigenetic memory

## Abstract

SARS-CoV-2 infection has raised significant concerns regarding its impact on assisted reproductive technology. We found that oocyte retrieval during acute SARS-CoV-2 infection significantly reduced the rates of good-quality blastocyst formation, but the underlying molecular mechanisms remain poorly understood. To address this, we investigated the effects of maternal acute SARS-CoV-2 infection on preimplantation embryo development and the early offspring hematopoietic system. Using single-cell RNA sequencing (scRNA-seq), we identified developmental delays in morphologically normal blastocysts from infected mothers, characterized by prolonged expression of zygotic genome activation-related genes, downregulation of mTORC1 signaling, and altered energy metabolism, including suppressed oxidative phosphorylation (OXPHOS) and enhanced glycolysis. We further revealed that maternal acute infection induced abnormal methylation/demethylation patterns in preimplantation embryos. To assess the potential long-term impact on offspring, we conducted integrated multi-tissue analyses, including bulk RNA-seq and genome-wide DNA methylation profiling of placental tissues, along with scRNA-seq of umbilical cord blood (UCB) cells from neonates delivered by SARS-CoV-2-infected mothers. Neonates exhibited elevated levels of inflammatory cytokines and an increased abundance of monocytes, indicating an activated myelopoiesis response. In addition, hematopoietic stem and progenitor cells (HSPCs) from UCB showed reduced OXPHOS activity and a skewed differentiation bias toward the myeloid lineage, potentially impacting long-term immune function. Collectively, these findings reveal that maternal acute SARS-CoV-2 infection impairs preimplantation embryo development and leaves a lasting imprint on offspring hematopoietic health through dysregulated energy metabolism, epigenetic modifications, and altered immune responses.

## Introduction

The COVID-19 pandemic, caused by SARS-CoV-2, has had profound global health implications, particularly in the field of reproductive medicine. Chronic inflammation, mediated by pro-inflammatory cytokines, is known to impair oocyte quality, thereby reducing the success rates of women undergoing assisted reproductive technology (ART) treatment^1^. Acute SARS-CoV-2 infection, which can even trigger a systemic cytokine storm^2^, poses unprecedented challenges for ART, particularly with regard to the safety and efficacy of oocyte retrieval and embryo transfer in infected women^3^. Although some studies have reported that SARS-CoV-2 RNA is undetectable in ovarian tissue, follicular fluid, cumulus cells, endometrial tissue, or vaginal fluid samples^4–6^, the potential for viral infection of oocytes remains a concern owing to the expression of the SARS-CoV-2 entry receptors, angiotensin-converting enzyme 2 (*ACE2*) and transmembrane serine protease 2 (*TMPRSS2*) in mature oocytes^7^.

Although a history of asymptomatic or mild SARS-CoV-2 infection does not adversely affect ART outcomes in fresh and frozen embryo transfer cycles^8^, acute SARS-CoV-2 infection has been associated with significantly lower rates of top-quality embryos, blastocysts, and blastocyst formation^9^. Another study involving women with a time interval from infection to oocyte retrieval of less than 7 days showed a significant decrease in oocyte utilization rate and a declining trend in other outcome indicators^10^. These studies suggest that acute infection during oocyte retrieval may impair embryo development and offspring health, although the specific molecular mechanisms remain poorly understood.

Environmental exposures, including those induced by inflammatory conditions, are closely linked to epigenetic reprogramming^11^, suggesting that such exposures can induce changes in epigenetic modifications, potentially affecting oocyte and preimplantation embryo development. Epidemiological evidence further indicates that environmental exposures can be transmitted across generations, with gametes likely playing a central role in this intergenerational transfer. For example, a study in mice has shown that paternal exposure to *Candida albicans* infection can lead to the transmission of inflammatory responses in their offspring^12^, although the phenotypic outcomes remain debated^13^. In addition, recent research has demonstrated that spermatozoa carry environmentally sensitive small non-coding RNAs, including mitochondrial tRNA fragments, that are influenced by paternal diet and mitochondrial dysfunction, and these RNAs are transferred to the oocyte during fertilization, affecting offspring metabolism and increasing the risk of obesity^14^. Similarly, maternal hyperglycemia has been shown to reduce the expression of *TET3* in oocytes, impairing DNA demethylation in zygotes and leading to metabolic disorders in adult offspring^15^. These findings collectively highlight the critical role of environmental exposures, including infections and metabolic stressors, in epigenetically altering germ cells and influencing offspring health. This aligns with the Developmental Origins of Health and Disease (DOHaD) theory, which posits that many adult diseases are closely linked to adverse environmental exposures during embryonic or gamete development^16^. By understanding how these exposures shape epigenetic modifications in germ cells, we can gain deeper insights into the mechanisms underlying intergenerational health effects and potentially develop strategies to mitigate their impact.

Inflammatory environment exposures can also influence the epigenetic modifications of many cell types, including hematopoietic stem and progenitor cells (HSPCs). For instance, systemic inflammation caused by SARS-CoV-2 infection in human adults causes persistent changes in the innate immune phenotype and epigenetics of HSPCs, which last months to a year^17^. These changes are associated with altered transcription factor (TF) activity, modified inflammatory responses, and increased myeloid cell bias^17^. Similarly, Bacillus Calmette–Guérin (BCG) vaccination induces epigenetic changes in human HSPCs that affect myeloid cell fate determination in the bone marrow for at least three months post vaccination^18^. In addition, maternal Zika virus infection during pregnancy has been found to cause developmental abnormalities in fetal HSPCs^19^. However, current research on inflammatory environment exposures and HSPCs is focused primarily on adult HSPCs or intrauterine infections, leaving a gap in our understanding of how inflammatory exposures of germ cells affect HSPCs in human offspring.

Research on embryo-origin HSPCs provides molecular-level insights into short- to long-term systemic immune function in adults, offering a unique perspective for understanding neonatal tissue and systemic immunity. Therefore, investigating whether abnormal environmental exposures induced by pathogen infections are “memorized” by oocytes and indirectly affect offspring health is crucial for optimizing ART clinical treatment strategies.

This study aimed to evaluate the effects of maternal acute SARS-CoV-2 infection on preimplantation embryo development and the early offspring hematopoietic system. We hypothesized that SARS-CoV-2 infection during oocyte retrieval might impair embryo quality and alter neonatal immune responses. By analyzing embryo development and neonatal immune profiles, we sought to provide insights into the molecular and cellular mechanisms underlying these effects. Our findings will contribute to the growing body of knowledge on the reproductive implications of acute infection and inform clinical decisions regarding ART during the pandemic.

## Results

### Overall design of this study

**Fig. 1:**
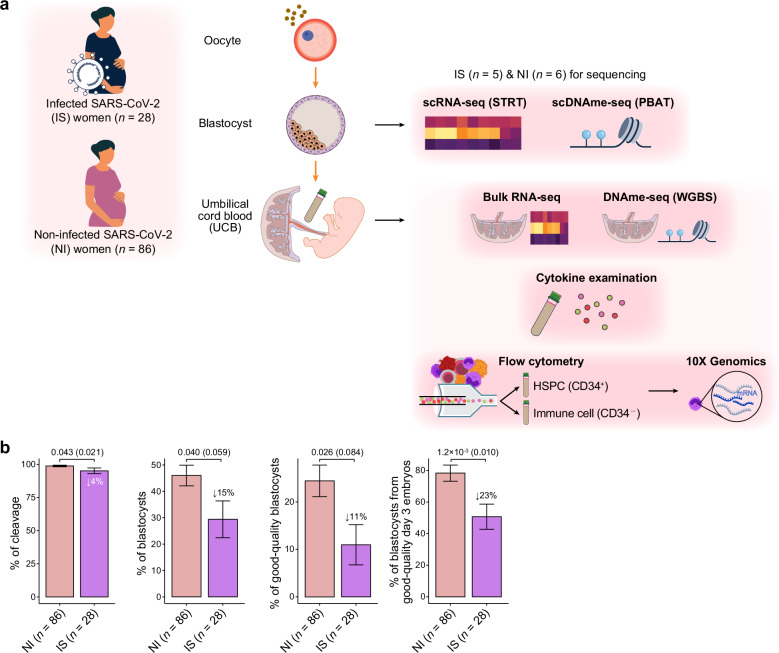
Reduced good-quality blastocyst formation due to maternal acute SARS-CoV-2 infection. **a** Schema showing the overall design of this study. **b** Barplots showing the percentages of cleavage, blastocysts, good-quality blastocysts, and blastocysts from good-quality day 3 embryos. *P*-values from two-tailed Wilcoxon rank sum tests are indicated. *P*-values from two-tailed Student’s *t*-tests after removal of the confounding factor of age by linear regression are indicated in parentheses, and percentage decreases are also indicated. Data are shown as mean ± SEM.

From December 17, 2022, to January 6, 2023, a total of 123 patients underwent ovarian stimulation cycles in our ART clinic. Among these, 28 patients were assigned to the SARS-CoV-2-positive (SARS-CoV-2 infected, IS) group, defined by a positive nucleic acid test or antigen test for SARS-CoV-2 within 24 h prior to oocyte retrieval and a body temperature below 37 °C during the procedure. The remaining 95 patients were initially classified as non-infected; however, 9 were excluded due to unsuccessful oocyte retrieval or cryopreservation, resulting in 86 patients in the SARS-CoV-2-negative group (non-infected, NI) (Fig. 1a). All participants in the IS and NI groups underwent either intracytoplasmic sperm injection (ICSI) or in vitro fertilization (IVF).

Good-quality blastocysts were transferred back into the maternal uterus to achieve pregnancy at least two months after oocyte retrieval, by which time the maternal body was no longer in the acute infection phase of SARS-CoV-2. In other words, maternal inflammatory status had resolved by the time of embryo transfer. Additional blastocysts were cryopreserved for future use. With the patients’ informed consent, the cryopreserved blastocysts, placental tissues, and neonatal umbilical cord blood (UCB) were collected for experimental purposes in this study (Fig. 1a). Placental tissues, UCB samples, and blastocysts were paired together in the IS group.

### Reduced good-quality blastocyst formation due to maternal acute SARS-CoV-2 infection

We systematically recorded key outcomes related to oocyte and embryo quality in 28 and 86 patients from the IS and NI groups, respectively. Baseline characteristics were comparable between the IS and NI groups (Supplementary Table S1). No significant differences were observed in oocyte-related outcomes, including the number of retrieved oocytes, oocyte maturation rate, normal fertilization rate, or number of bipronuclear zygotes (2PN) (Supplementary Table S1). Similarly, embryo development indicators, such as the number and rate of good-quality embryos on day 3, the number of blastocysts and good-quality blastocysts, and the oocyte utilization rate, did not differ significantly between the two groups (Supplementary Table S1). However, the IS group exhibited significantly lower rates of cleavage (100% (IQR 100%–100%) vs 100% (100%–100%), two-tailed Wilcoxon rank sum, *P* = 0.043), blastocyst formation (20% (0%–54%) vs 50% (15%–67%), *P* = 0.040), good-quality blastocyst formation (0% (0%–12%) vs 18% (0%–33%), *P* = 0.026), and blastocyst formation from good-quality day 3 embryos (50% (40%–67%) vs 100% (67%–100%), *P* = 0.001) compared to the NI group (Fig. 1b; Supplementary Table S1). After adjustment for age-related effects, we observed a 4% reduction in the rate of cleavage (two-tailed Student’s *t*-test, *P* = 0.02) and a 23% decline in the rate of blastocysts from good-quality day 3 embryos (*P* = 0.01) in the IS group compared to the NI group (Fig. 1b).

This finding suggests that maternal acute SARS-CoV-2 infection adversely affects early embryonic development independently of maternal age, consistent with previous studies of larger cohorts^9,10^. In addition, no significant differences were observed in the number of cleavages, available day 3 embryos, available blastocysts, or rate of available day 3 embryos or good-quality blastocysts from good-quality day 3 embryos between the groups (Supplementary Table S1).

Among the 28 enrolled couples in the IS group, our analysis identified 8 patients whose male partners tested positive for SARS-CoV-2 infection on the oocyte retrieval day. However, the subgroup comparison revealed no statistically significant differences in measured oocyte or embryo developmental parameters based on paternal infection status (Wilcoxon rank sum test or *χ*^2^ test, *P* > 0.05) (Supplementary Table S1). Notably, all blastocyst, placental tissue, and UCB samples collected for sequencing were obtained from women whose male partners tested negative for SARS-CoV-2 infection.

### Transcriptome evidence for developmental delay in IS good-quality blastocysts

Although good-quality blastocysts from the IS group exhibited normal morphological phenotypes, their molecular characteristics were unexplored. We therefore performed single-cell RNA sequencing (scRNA-seq) on five blastocysts from NI donors and three blastocysts from IS donors using a modified single-cell tagged reverse transcription (STRT) technique^20,21^ (Fig. 1a; Supplementary Fig. S1a). After stringent quality control, high-quality transcriptomes from 224 single cells collected from the IS and NI groups were retained for subsequent analysis (Supplementary Fig. S1b). Based on the expression levels of X and Y chromosome genes, NI_Embryo8 was identified as male, and the remaining embryos were female (Supplementary Fig. S1c).

**Fig. 2:**
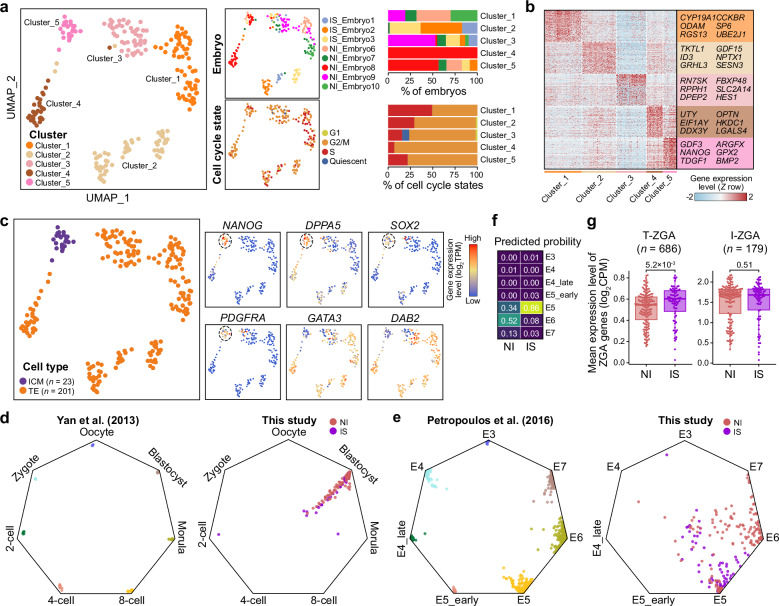
scRNA-seq data from blastocysts provide molecular evidence of developmental delay in good-quality blastocysts from the IS group. **a** Left, UMAP plots showing scRNA-seq data from blastocysts colored according to unsupervised clustering, embryos, and cell cycle status. Right, stacked barplots showing the percentage of distinct embryos (top) and cell cycle status (bottom) in each cluster. **b** Heatmap showing the expression profiles of differentially expressed genes (DEGs) among clusters. Representative DEGs are listed at right. **c** Left, UMAP plot showing scRNA-seq data colored by cell type. Right, UMAP plots showing the expression profiles of well-known marker genes of ICM or TE cells. **d**, **e** Scatter plots showing the distribution of single cells in the established machine-learning model. Single cells were collected from public scRNA-seq data (left) and from this study (right). The public scRNA-seq data define the oocyte and preimplantation embryo at distinct stages (**d**) or distinct developmental days (**e**). **f** Heatmap showing the probability of single cells being assigned to specific developmental days, as calculated by the established machine-learning model, corresponding to **e**. **g** Boxplots showing the expression levels of ZGA-related (T-ZGA and I-ZGA) genes in blastocysts from NI and IS groups. The numbers of T-ZGA- or I-ZGA-related genes are indicated in brackets. *P*-values from one-tailed Wilcoxon rank sum tests are indicated.

Unsupervised clustering and uniform manifold approximation and projection (UMAP) visualization revealed five distinct cell clusters (Fig. 2a, left), each characterized by unique gene expression profiles (Fig. 2b). Cells were primarily segregated by donor group, cell cycle state, and cell type. Cluster_1 consisted of cells from NI donors, whereas cluster_2 was enriched for cells from IS donors (Fig. 2a, right). Cluster_3 mainly comprised cells in the G2/M and quiescent states, and cluster_4 contained cells exclusively from the male NI_Embryo8 (Fig. 2a; Supplementary Fig. S1c). Cluster_5 was identified as inner cell mass (ICM) cells expressing ICM-specific marker genes, such as *NANOG*, *DPPA5*, *SOX2*, and *PDGFRA* (Fig. 2c). The remaining clusters (except for cluster_5) were identified as trophectoderm (TE) cells expressing TE-specific marker genes, including *GATA3* and *DAB2* (Fig. 2c). To determine whether ICM cells isolated in this study could be further differentiated into primitive endoderm (PE) and epiblast (EPI) lineages, we analyzed the expression levels of canonical lineage-specific marker genes (*NANOG* for EPI and *GATA4* for PE). Among the 23 ICM cells examined, only two cells exhibited PE lineage characteristics as indicated by *GATA4* expression, whereas most cells demonstrated EPI lineage identity as indicated by *NANOG* expression (Supplementary Fig. S2a).

We detected a developmental fault in blastocyst formation in the IS group (Fig. 1b). To further investigate the existence of developmental delays in good-quality blastocysts from the IS group at the molecular level, we developed a machine-learning model to compare our single-cell transcriptomes with publicly available datasets. This model enabled visualization of each single cell based on the probability of its belonging to each of the prototypical cell types. We first established the model using public scRNA-seq data that defined different human preimplantation embryo stages^22^. Our model accurately classified public data into developmental stages and grouped our single cells into the ‘blastocyst’ cluster (Fig. 2d). We then developed a second model, trained on public scRNA-seq data that defined the developmental days of human preimplantation embryos^23^, and applied this model to our scRNA-seq data (Fig. 2e, left). In the NI group, 34% and 52% of the cells were classified as day 5 and 6 blastocysts, respectively, consistent with their collection timing and supporting the accuracy of our established model (Fig. 2f; Supplementary Figs. S1a, S2b). By contrast, 86% and 8% of cells in the IS group were classified as day 5 and 6 blastocysts, respectively, despite 58% of these cells (from IS_Embryo1 and IS_Embryo3) being collected from day 6 blastocysts (Fig. 2f; Supplementary Figs. S1a, S2b).

We next analyzed the expression of zygotic genome activation (ZGA)-related genes, which were identified based on public scRNA-seq data^22^. We classified ZGA-related genes into two groups based on previously reported patterns (see “Materials and methods” section for details)^24^: “transient ZGA (T-ZGA, *n* = 686)”, comprising genes that peak in expression at the 8-cell stage and then decline at the morula and blastocyst stages; and “increasing ZGA (I-ZGA, *n* = 179)”, comprising genes that show continuous upregulation after activation. Typically, T-ZGA-related gene expression gradually declines from the 8-cell stage to blastocyst formation (Supplementary Fig. S2c). However, we observed that the expression levels of T-ZGA-related genes were significantly elevated in IS cells compared to NI cells (one-tailed Wilcoxon rank sum, *P* = 5.3 × 10^–3^) (Fig. 2g; Supplementary Fig. S2d). By contrast, I-ZGA-related gene expression did not differ significantly between IS and NI cells (*P* = 0.51) (Fig. 2g; Supplementary Fig. S2d). These findings suggest that the repression of T-ZGA-related genes, which normally occurs at appropriate embryonic stages, was impaired in IS blastocysts.

Collectively, these findings suggest that despite their normal morphological appearance, good-quality blastocysts generated from SARS-CoV-2-infected mothers exhibited molecular profiles of developmental delay, including prolonged ZGA-related gene expression and slower progression through preimplantation stages. This highlights the potential impact of maternal acute SARS-CoV-2 infection on early embryonic development at the transcriptomic level.

### Abnormal mTORC1 signaling and energy metabolism in IS good-quality blastocysts

**Fig. 3:**
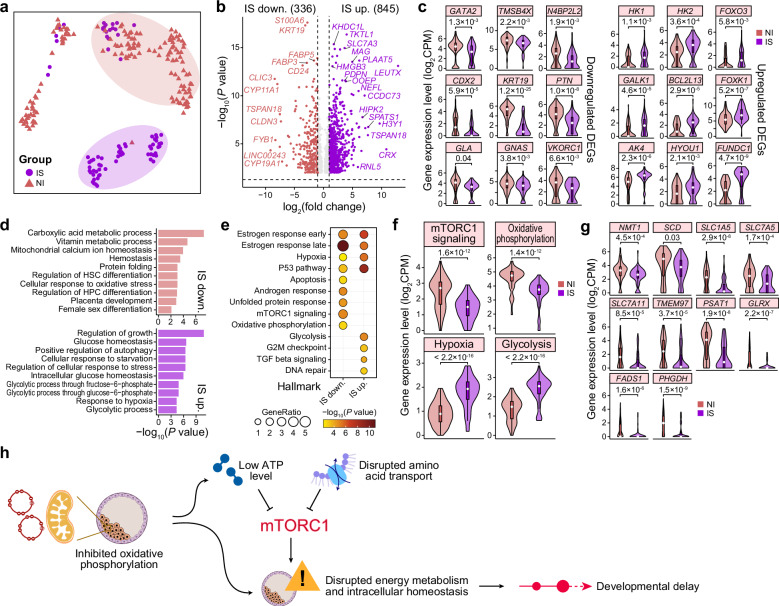
scRNA-seq data from blastocysts reveal altered mTORC1 signaling and energy metabolism in good-quality blastocysts from the IS group. **a** UMAP plot showing scRNA-seq data from blastocysts colored according to the group (IS or NI). **b** Volcano plot showing upregulated (purple text) and downregulated (pink text) DEGs in IS blastocysts compared to NI blastocysts. The numbers of DEGs are indicated in parentheses. Representative DEGs are listed. **c** Violin plots showing the expression levels of representative downregulated (left) and upregulated (right) DEGs in blastocysts between the IS and NI groups. *P*-values from two-tailed Student’s *t*-tests are indicated. **d** Barplots showing GO terms (biological process) corresponding to downregulated (top) and upregulated (bottom) DEGs in IS blastocysts compared to NI blastocysts. **e** Dot plot showing GO terms (hallmark) corresponding to downregulated (top) and upregulated (bottom) DEGs in IS blastocysts compared to NI blastocysts. Dot size indicates the ratio of related genes among inputs, and dot color indicates statistical significance. **f** Violin plots showing the average expression levels of representative hallmark-related genes. *P*-values from two-tailed Student’s *t*-tests are indicated. **g** Violin plots showing the expression levels of representative genes related to mTORC1 signaling. *P*-values from two-tailed Student’s *t*-tests are indicated. **h** Schema illustrating the hypothesized disruption of energy metabolism and cellular homeostasis in IS blastocysts.

The UMAP visualization revealed distinct transcriptomic profiles between the IS and NI groups (Fig. 3a). To decipher potential factors driving the developmental delay of IS blastocysts in detail, we identified differentially expressed genes (DEGs) between TE cells from the IS and NI groups, excluding the sole male embryo (NI_Embryo8). We identified 845 upregulated and 336 downregulated DEGs in the IS group, reflecting a dramatic transcriptomic difference between the two groups (Fig. 3b; Supplementary Table S2). We detected a significant overlap between the upregulated DEGs and T-ZGA-related genes (Fisher’s exact test, *P* = 3.6 × 10^–10^), supporting the developmental delay of embryos in the IS group (Supplementary Fig. S2e).

Gene Ontology (GO) analysis revealed that downregulated DEGs were related to hemostasis (e.g., *GLA*, *GNAS*, and *VKORC1*), protein folding (e.g., *HSPA1A*, *HSP90AA1*, and *DNAJC1*), placenta development (e.g., *CDX2*, *KRT19*, and *PTN*), and female sex differentiation (e.g., *LRP2* and *UBB*) (Fig. 3b, c; Supplementary Fig. S2f and Table S2). We found that downregulated DEGs were associated with mitochondrial calcium ion homeostasis (e.g., *ANXA6* and *TGM2*) (Fig. 3c, d) and hallmarks of oxidative phosphorylation (OXPHOS) (e.g., *GPX4*, *ATP5PD*, *UQCR11*, and *NDUFS7*) (Fig. 3e, f; Supplementary Fig. S2f), suggesting that mitochondrial function may be inhibited in IS blastocysts. Consistent with this notion, ATP-dependent protein folding chaperone-related genes, including *HSPA1A*, *HSPA1B*, *HSP90AA1*, and *TRAP1*, were downregulated in the IS group (Supplementary Table S2). The observed transcriptional differences were not attributable to embryo-to-embryo variation (Supplementary Fig. S2h).

Importantly, we found that downregulated DEGs were associated with mechanistic target of rapamycin (mTOR) complex 1 (mTORC1) signaling (Fig. 3e–g; Supplementary Fig. S2h). The mTOR signaling pathway, a key regulator of cellular metabolism, plays an indispensable role during early mammalian embryogenesis^25,26^. Consistent with our findings, a previous study demonstrated that mTOR suppression during the preimplantation period reduces blastocyst formation rates and implantation competency, ultimately impairing post-implantation embryonic development^27^. Recent research has revealed that attenuated activity of the mTOR signaling pathway induces blastoids to enter a dormant state characterized by restricted proliferation, arrested developmental progression, and diminished capacity for endometrial cell attachment^28^. mTORC1, as one of two multi-molecular complexes, regulates the balance between catabolism and anabolism by integrating information about nutritional abundance and environmental status^26^. We found that the expression of genes encoding the cell surface transporters SLC1A5 and SLC7A5, which regulate the transport of L-glutamine and L-leucine to activate mTOR^29^, was lower in the IS group (Fig. 3g), and *PHGDH* and *PAST1* were also downregulated in the IS group (Fig. 3g). The expression of *PHGDH* and *PAST1* is associated with activation of serine and one-carbon metabolism by mTORC1 signaling^30^, and cellular serine levels in turn influence mTORC1 activation^31,32^.

The IS group showed upregulation of genes related to glucose homeostasis (e.g., *HK1*, *HK2*, *FOXO1*, *FOXO3*, *INSR*, and *IGF1R*), the glycolytic process (e.g., *ENO2*, *GALK1*, and *PGAM4*), and response to hypoxia (e.g., *AK4*, *HYOU1*, *TGFBR3*, and *FUNDC1*) (Fig. 3c, d; Supplementary Fig. S2g and Table S2). Genes related to the hallmarks of glycolysis and hypoxia were also significantly upregulated in the IS group compared to the NI group (Fig. 3e, f; Supplementary Fig. S2h). Incompatible stresses, including hypoxia, low ATP levels, DNA damage, and reduced amino acid transport, have been reported to induce the inhibition of mTORC1 to disrupt cellular energy metabolism and intracellular homeostasis^33–36^. A previous study has also demonstrated that inhibition of mTORC1 reduces mitochondrial activity and biogenesis^37^. On the basis of these findings, we inferred that inhibited mitochondrial function may not generate sufficient energy to support the development of preimplantation embryos. At the same time, low ATP levels and limited amino acid transport may inhibit mTORC1 signaling, further disrupting cellular energy metabolism and intracellular homeostasis (Fig. 3h). Alternatively, inhibited mitochondrial function may directly promote the disruption of cellular energy metabolism.

### DNA hypermethylation profiles support the developmental delay of IS blastocysts

**Fig. 4:**
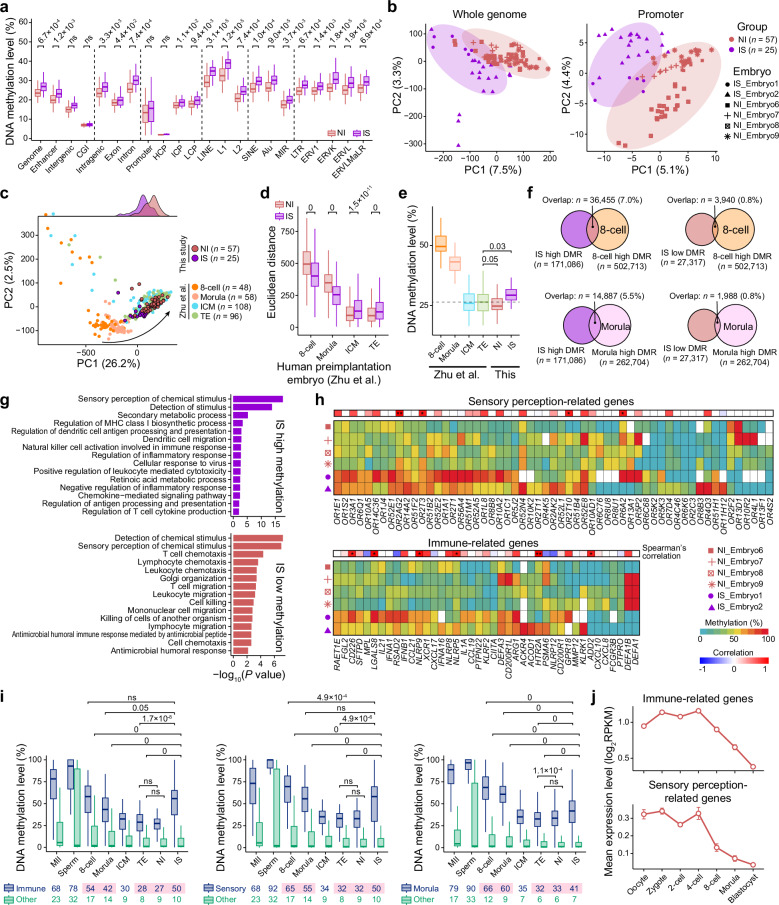
Single-cell DNA methylation data from blastocysts reveal abnormal methylation/demethylation patterns in IS blastocysts. **a** Boxplot showing the average DNA methylation levels of different genomic elements in blastocysts from the IS and NI groups. The average DNA methylation level of each single cell was calculated. *P*-values from two-tailed Student’s *t*-tests are indicated. **b** PCA plots showing the cell distribution based on DNA methylation profiles of the whole genome (left) and promoters (right). The numbers of single cells are indicated in parentheses. **c** PCA plot showing the cell distribution based on whole-genome DNA methylation profiles, including data from our study and publicly available human preimplantation embryos. The cellular density distributions of blastocysts from the IS and NI groups are shown at the top. The arrow indicates the developmental trajectory of human preimplantation embryos. The numbers of single cells are indicated in parentheses. **d** Boxplot showing the Euclidean distance within the PCA space between IS/NI blastocysts and publicly available human preimplantation embryos. *P*-values from two-tailed Student’s *t*-tests are indicated. **e** Boxplot showing the average whole-genome DNA methylation levels in blastocysts from the IS/NI groups and publicly available human preimplantation embryos. The average DNA methylation level of each single cell was calculated. The statistical significance of differences between publicly available TE cells and IS/NI blastocysts was assessed. *P*-values from two-tailed Student’s *t*-tests are indicated. The dashed line indicates the median DNA methylation level of the publicly available TE cells. **f** Venn diagrams showing the overlap between IS DMRs (left, hypermethylated DMRs; right, hypomethylated DMRs) and embryo stage-specific hypermethylated DMRs (top, 8-cell embryo; bottom, morula). The overlap percentages and numbers of embryo stage-specific hypermethylated DMRs are indicated. **g** Barplots showing GO terms corresponding to genes with hypermethylated (top) and hypomethylated (bottom) promoters in IS blastocysts compared to NI blastocysts. **h** Heatmaps showing the average DNA methylation levels of promoters related to sensory perception (top) and immunity (bottom). Spearman’s correlation analysis was performed between promoter methylation and expression levels, and the coefficients and corresponding *P*-values are indicated at the top. Each row corresponds to one embryo. *P < 0.1 and **P < 0.01. **i** Boxplots showing the average DNA methylation levels of immune-related promoters (left; *n* = 57), sensory perception-related promoters (middle; *n* = 64), morula-specific DMPs excluding immune- and sensory perception-related genes (right; *n* = 2248), and other promoters in IS/NI blastocysts and publicly available human preimplantation embryos. The average DNA methylation levels of the genes are indicated at the bottom. *P*-values from two-tailed Student’s *t*-tests are indicated. **j** Line plots showing the average expression levels of immune-related (top) and sensory perception-related genes (bottom) across human preimplantation development. The expression data were collected from a previous study. Data are shown as mean ± SEM.

To investigate whether there were epigenetic differences between blastocysts from the IS and NI groups, we performed post-bisulfite adapter tagging (PBAT) DNA methylome analysis of human blastocysts at single-cell and single-base resolution (Fig. 1a). For 82 high-quality single cells (25 and 57 cells from the IS and NI groups, respectively), our PBAT DNA methylation data covered 5.7 million CpG sites (≥ 1×) (Supplementary Fig. S3a and Table S3). Both the whole genome and subgenomic elements exhibited higher DNA methylation levels in the IS group than in the NI group, in line with the transcriptomic phenotype of developmental delays in IS blastocysts (Figs. 2e, 4a). Consistent with this finding, a previous study revealed that mTOR suppression increases global DNA methylation in blastocysts^27^. Moreover, a principal component analysis (PCA) showed differences in DNA methylation between the two groups across the whole genome (Fig. 4b, left).

To further examine the epigenetic aspects of this developmental delay, we obtained previously published single-cell DNA methylation data from human preimplantation embryos^38^ and compared them with our data. We first analyzed global DNA methylation patterns using PCA. The developmental trajectory along the first principal component 1 (PC1) axis clearly distinguished the 8-cell embryo, morula, and blastocyst lineages (ICM and TE cells) (Fig. 4c). Whereas NI blastocysts clustered near ICM/TE cells (as expected for their developmental stage), those from the IS group were consistently localized closer to morula cells (Fig. 4c). Quantitative analysis of Euclidean distances within the PCA space confirmed this observation: IS blastocysts showed significantly shorter distances from 8-cell embryos (two-tailed Student’s *t*-test, *P* = 0) and morulae (*P* = 0) but greater distances from ICM/TE cells (*P* = 1.5 × 10^–11^ and 0, respectively), compared to the NI group (Fig. 4d). Moreover, the DNA methylation level of the whole genome was significantly higher in IS blastocysts than in TE cells (*P* = 0.03) (Fig. 4e). These results suggest that IS blastocysts exhibited a morula-like global DNA methylation profile.

There were three outlier blastocysts from the IS group in the PCA space (Fig. 4b; Supplementary Fig. S3b). Compared to other IS blastocysts, these outliers exhibited significantly higher global DNA methylation levels (two-tailed Student’s *t*-test, *P* = 1.8 × 10^–9^) (Supplementary Fig. S3c, left). Moreover, when we observed the Euclidean distances within the PCA space that included our blastocysts and publicly available human preimplantation embryos (Fig. 4c), the outlier blastocysts tended to be closer to 8-cell embryos (*P* = 0.45) and morula cells (*P* = 0.25) but farther from ICM/TE cells (*P* = 0.65 and 0.65, respectively), compared to other IS blastocysts (Supplementary Fig. S3c, right).

Given the critical role of imprinted genes in placental development and fetal growth regulation, we assessed DNA methylation levels at known human imprinting control regions (ICRs)^39^. In blastocysts from both the IS and NI groups, most ICRs exhibited comparable average methylation levels (~50%), consistent with published human blastocyst data^38^ (Supplementary Fig. S3d). PCA of ICR methylation profiles also revealed no separation between the IS and NI groups (Supplementary Fig. S3e). Notably, the ICR *RB1* showed pronounced hypermethylation in IS blastocysts (69.0% vs 30.3% in the NI group; two-tailed Student’s *t*-test, *P* = 4.6 × 10^–111^). Since *RB1* inactivation promotes excessive trophoblast proliferation in mice^40^, this aberrant methylation may disrupt placental function in the IS group. Similarly, the ICR *PEG10*, which is essential for placental formation and placental vasculature maintenance^41^, showed significantly higher methylation in the IS group (37% vs 26% in the NI group, *P* = 2.0 × 10^–8^). Partial retention of maternal allele-specific methylation was also observed at other ICRs, including *DIRAS3* and *NAP1L5*. These findings suggest that although most parental allele-specific DNA methylation patterns were largely erased in IS blastocysts, certain maternal ICRs retained partial methylation patterns, potentially influencing placental development in the IS group.

We identified 27,317 differentially methylated regions (DMRs) with lower methylation levels (≤ 60%) and 171,086 DMRs with higher methylation levels (≥ 60%) in the IS group compared to the NI group, and these DMRs were primarily distributed in intergenic regions, as expected (Supplementary Fig. S3f). These results indicated a dramatic difference in DNA methylation between the two groups. We also identified 502,713 stage-specific DMRs in 8-cell embryos relative to TE cells using public human preimplantation embryo data^38^. Notably, IS blastocysts exhibited a higher proportion of hypermethylated DMRs overlapping with 8-cell DMRs (7.0%) compared to hypomethylated DMRs (0.8%) (Fig. 4f, top). This methylation pattern bias was also observed in morula-stage DMRs (Fig. 4f, bottom).

Collectively, our DNA methylation analyses demonstrated that IS blastocysts exhibited global methylation profiles resembling those of earlier developmental stages, and these blastocysts retained more DMRs associated with 8-cell and morula embryos, further supporting the developmental delay.

### Abnormal DNA methylation patterns in immune- and sensory perception-related genes within IS blastocysts

We also detected distinct methylation profiles in promoter regions, with 172 and 817 differentially methylated promoters (DMPs) exhibiting lower and higher methylation levels in the IS group, respectively (Fig. 4b, right; Supplementary Table S3). GO analysis demonstrated that highly methylated promoters were enriched in sensory perception of chemical stimulus (e.g., *OR3A1* and *OR1E1*), regulation of MHC class I biosynthetic process (e.g., *IFNB1*, *CIITA*, and *NLRP12*), regulation of inflammatory response (e.g., *CXCL17*, *IL21*, and *NRRP8*), and cellular response to virus (e.g., *IFNA1*, *IFNA16*, and *CCL19*) (Fig. 4g, h). We also detected a limited number of weakly methylated promoters in the IS group, including *FCGR3B*, *CXCL8*, *CXCL10*, *DEFA1*, and *DEFA1B*, which are related to cell killing or cell chemotaxis (Fig. 4g, h).

To systematically examine the relationship between promoter DNA methylation and gene expression level, we performed Spearman’s correlation analysis using publicly available single-cell data for human preimplantation embryos, combining RNA-seq^22^ and DNA methylation data^38^ across developmental stages. Notably, most immune- and sensory perception-related genes exhibited a non-significant positive correlation (Spearman’s correlation coefficient *ρ* > 0, *P* > 0.1) between promoter methylation and expression level (Fig. 4h). Correspondingly, and consistent with the observed promoter DNA methylation dynamics (Fig. 4i, left and middle), these genes were progressively downregulated during development (Fig. 4j), demonstrating coordinated epigenetic and transcriptional regulation. In blastocysts, these genes were effectively silenced (expression < 1) (Fig. 4j), with no significant differential expression observed between the IS and NI groups (Supplementary Fig. S3g), although differential methylation was detected.

We next investigated whether maternal acute SARS-CoV-2 infection altered demethylation patterns within specific genomic regions during preimplantation development using the publicly available human embryonic dataset^38^. We first identified DMPs with higher methylation levels in morula cells compared with TE cells. After excluding immune- and sensory perception-related genes (*n* = 120) from the morula-specific DMPs (*n* = 2298), we retained 2248 DMPs for downstream analysis. The DNA methylation levels of these morula-specific DMPs decreased progressively during preimplantation development (Fig. 4i, right). Notably, within the morula-specific DMPs, NI blastocysts exhibited methylation levels similar to those of TE cells (33% vs 32%), whereas IS blastocysts showed methylation intermediate (41%) between that of TE cells (32%) and morula cells (60%) (Fig. 4i, right). This observation aligned with global DNA methylation patterns, which showed that IS blastocysts had a morula-like DNA methylation profile (Fig. 4e). The promoter methylation levels of immune-related genes in IS blastocysts (50%) closely resembled those in 8-cell embryos (54%, two-tailed Student’s *t*-test, *P* = 0.34) but were significantly higher than those in morulae (42%, *P* = 0.05) (Fig. 4i, left). Sensory perception-related genes in IS blastocysts (50%) displayed promoter methylation levels significantly lower than those of 8-cell embryos (65%, *P* = 4.9 × 10^–4^) but comparable to those of morula (55%, *P* = 0.27) (Fig. 4i, middle). Thus, although IS blastocysts exhibited a morula-like global methylation profile, their immune-related gene promoters resembled those of 8-cell embryos, and their sensory perception-related gene promoters exhibited a morula-like profile.

These findings indicated that maternal acute SARS-CoV-2 infection might alter methylation/demethylation patterns in oocytes and subsequent preimplantation embryos, particularly in sensory perception- and immune-related genes.

### Placental transcriptomics reveals immune activation in the IS group

Given the critical role of TE cells in placental development, we investigated the transcriptional characteristics of IS placental tissues. Placental tissues and UCB samples (including serum and cells) were collected from eight neonates, four derived from oocyte retrieval from SARS-CoV-2-positive women and four born to women with no reported history of SARS-CoV-2 infection during pregnancy (Fig. 1a; Supplementary Table S4).

**Fig. 5:**
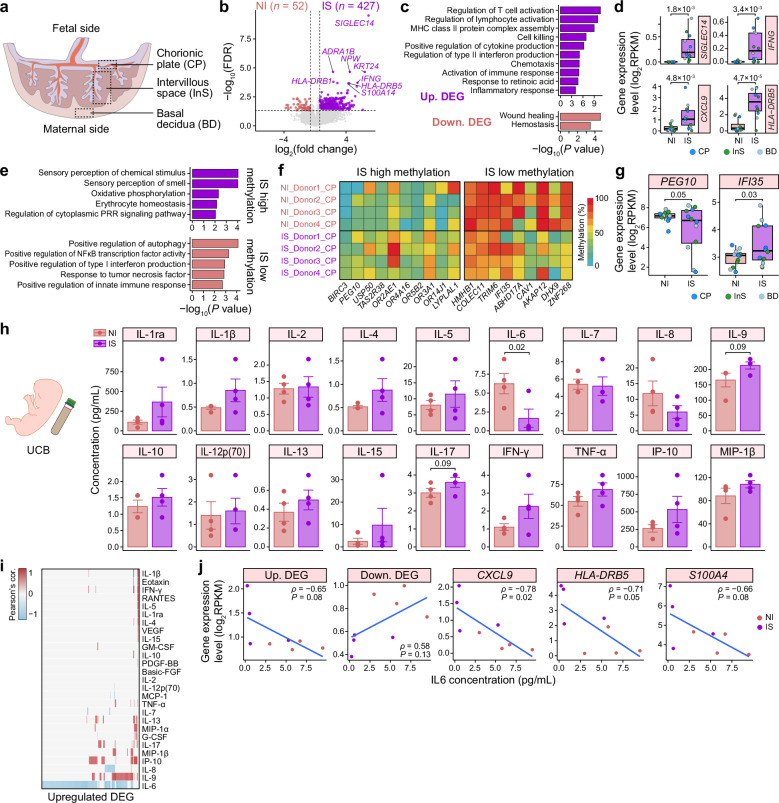
The IS placentas displayed immune response activation. **a** Cartoon showing the anatomical structures of the placenta used for bulk RNA-seq and DNA methylation profiling. **b** Volcano plot showing the distribution of DEGs between the IS and NI groups. Representative DEGs are indicated. The numbers of DEGs are indicated. **c** Barplot showing the GO terms associated with upregulated (top) and downregulated (bottom) DEGs in IS placentas compared to NI placentas. **d** Boxplots showing the expression levels of representative DEGs between the IS and NI groups. *P*-values from one-tailed Student’s *t*-tests are indicated. **e** Barplots showing GO terms associated with genes that had hypermethylated (top) and hypomethylated (bottom) promoters in IS placentas compared to NI placentas. **f** Heatmap showing the average DNA methylation levels of representative DMPs. Each row corresponds to a sample. **g** Boxplots showing the expression levels of *PEG10* and *IFI35* in placentas from the IS and NI groups. *P*-values from one-tailed Student’s *t*-tests are indicated. **h** Barplots showing the concentrations of representative cytokines in UCB serum. Each dot indicates one neonate. Data are shown as mean ± SEM. *P*-values from one-tailed Wilcoxon rank sum tests are indicated. **i** Heatmap showing Pearson’s correlation coefficients between the expression of individual upregulated DEGs in IS placentas and the cytokine concentrations in UCB serum. **j** Scatter plots showing Pearson’s correlations between the expression of (all or representative) upregulated/downregulated DEGs in IS placentas and the IL-6 concentration in UCB serum. Pearson’s correlation coefficients (*ρ*) and correlation test significance values (*P*) are indicated.

By performing bulk RNA-seq on three placental structures — chorionic plate (CP), intervillous space (InS), and basal decidua (BD) — we identified 427 upregulated and 52 downregulated DEGs in the IS group compared to the NI group (Fig. 5a, b; Supplementary Table S4). GO analysis revealed that upregulated DEGs were enriched in T/lymphocyte activation (i.e., *IFNG*, *LAG3*, and *TIGIT*), antigen processing and presentation (i.e., *HLA-DRB1* and *HLA-DRB5*), and inflammatory response (i.e., *CXCL9* and *S100A4*), whereas hemostasis-related genes (i.e., *PF4*, *THBD*, and *TUBB1*) were downregulated (Fig. 5c, d; Supplementary Fig. S4a). Notably, *IL6* expression was significantly reduced in the IS placenta, albeit modestly (two-tailed Student’s *t*-test, *P* = 0.04) (Supplementary Fig. S4a). In addition, compared to InS, CP and BD exhibited greater transcriptional differences between the IS and NI groups (Supplementary Fig. S4b). These findings suggested a pro-inflammatory placental microenvironment in the IS group.

Given the abnormal methylation/demethylation pattern in TE cells from the IS group, we further investigated placental DNA methylation characteristics in this group using whole-genome bisulfite sequencing (WGBS) of CP samples (Supplementary Table S4). Comparative analysis identified 196 DMPs with higher methylation in the IS group and 586 in the NI group (Supplementary Table S4). GO analysis revealed that hypermethylated DMPs in the IS group were associated with sensory perception (i.e., *OR2AE1*, *OR4A16*, and *OR5B2*), OXPHOS (i.e., *ATP5F1A*, *COX8A*, and *NDUFAB1*), and regulation of the cytoplasmic pattern recognition receptor (PRR) signaling pathway (i.e., *BIRC3*, *LYPLAL1*, and *USP50*) (Fig. 5e, f). Notably, the promoter methylation level of *PEG10* was significantly higher in the IS group (41% vs 25% in the NI group, two-tailed Student’s *t*-test, *P* = 1.7 × 10^–3^) (Fig. 5f), and *PEG10* was downregulated in IS placentas (Fig. 5g). This difference in *PEG10* methylation aligned with ICR patterns in blastocysts (Supplementary Fig. S3d). Furthermore, sensory perception-related genes exhibited persistent promoter hypermethylation in IS placentas, consistent with the observation in IS blastocysts (Fig. 4h). By contrast, hypomethylated promoters in the IS group were enriched for genes involved in positive regulation of autophagy (i.e., *GBA1* and *PIK3CB*), NFκB transcription factor activity (i.e., *CHUK* and *ROR1*), and the innate immune response (i.e., *CAV1* and *IFI35*) (Fig. 5e, f). This pattern was consistent with placental immune activation in the IS group, supported by significant upregulation of *IFI35* expression (Fig. 5g).

### Inflammatory cytokine concentrations exhibited an increased trend in the IS group

SARS-CoV-2 infection induces a cytokine storm in the systemic circulation of adults^2^. A previous study revealed that pro-inflammatory cytokine responses are present in the circulation of pregnant women with SARS-CoV-2 infection and their neonates^42^. To investigate whether neonates derived from oocytes of mothers with acute SARS-CoV-2 infection exhibited cytokine responses, we measured the concentrations of 27 cytokines in UCB serum (Fig. 1a). The IS group showed a slight increase in systemic concentrations of IL-9 (1.3-fold change (FC), one-tailed Wilcoxon rank sum, *P* = 0.09), IL-17 (1.2-FC, *P* = 0.09), and RANTES (CCL5) (1.5-FC, *P* = 0.09), but a significant decrease in IL-6 (3.7-FC, *P* = 0.02) compared to the NI group (Fig. 5h; Supplementary Fig. S4c). Notably, prior studies have reported no significant differences in maternal or neonatal serum IL-6 levels between: (1) mothers with recent/ongoing SARS-CoV-2 infection and those who had recovered^43^, or (2) historically infected and non-infected mothers^42^. Although these differences were not statistically significant, levels of pro-inflammatory cytokines, including IFN-γ (2.0-FC), TNF-α (1.3-FC), IP-10 (CXCL10) (2.0-FC), and MIP-1β (1.2-FC), tended to be higher in the IS group (Fig. 5h; Supplementary Fig. S4c). We also noted that levels of the anti-inflammatory factor IL-1ra (3.3-FC) tended to be higher in the IS group (Fig. 5h). These results suggested a slightly higher concentration of inflammatory cytokines in the IS group.

Pearson’s correlation analysis between placental upregulated DEGs in the IS group and UCB serum cytokine levels revealed that most upregulated DEGs, including *CXCL9*, *HLA-DRB5*, and *S100A4*, were negatively correlated with IL-6 concentration (Pearson’s *P* ≤ 0.1) (Fig. 5i, j). Despite the significant downregulation of *IL6* expression in the IS placenta and reduced IL-6 levels in IS UCB serum, no significant correlation was observed between them. These findings suggested that placental pro-inflammatory activation may suppress IL-6 levels in UCB serum, possibly because fetal compensatory mechanisms counteract the gamete-derived factor-induced inflammatory response in placentas.

### The scRNA-seq landscape of UCB cells and emergency myelopoiesis of activated monocytes in the IS group

The single-cell DNA methylation analysis above revealed hypermethylation of immune-related genes in IS blastocysts. However, it was not clear whether the function of HSPC differentiation and immune response was specific to the IS group. HSPCs, as precursors of immune cells, replenish peripheral needs to support hematopoiesis and immune responses; therefore, the molecular characteristics of HSPCs in neonates may influence their immune status in the long term. Thus, a comprehensive analysis of key gene expression regulation is required to understand the features of the human fetal immune system in neonates derived from IS oocytes.

To investigate the molecular features of immune cells and CD34^+^ HSPCs among UCB cells, we sorted UCB cells by flow cytometry and performed scRNA-seq using the 10× Genomics platform (Fig. 1a; Supplementary Fig. S5a). After stringent quality control, 127,748 high-quality single cells from ten scRNA-seq libraries were retained for subsequent analysis, with an average of 2256 detected genes, 6108 unique molecular identifiers (UMIs), and 3.6% mitochondrial transcripts per cell (Supplementary Fig. S5b).

**Fig. 6:**
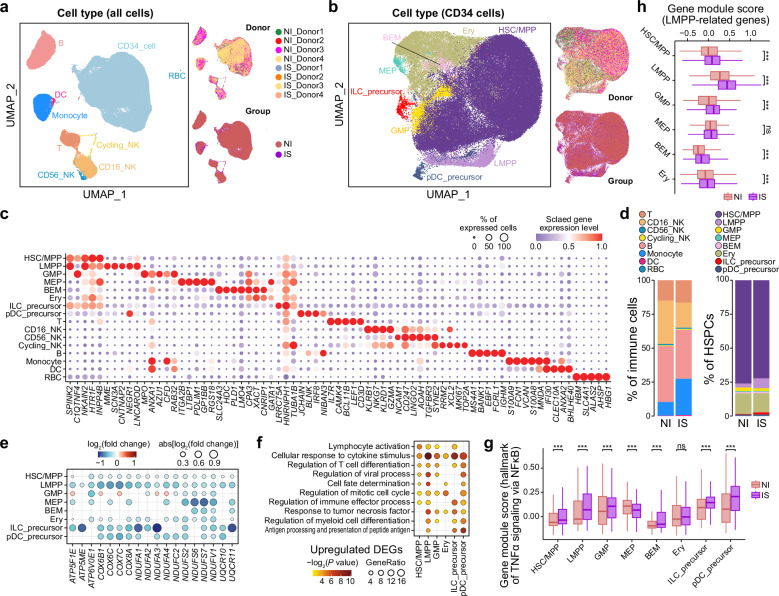
Single-cell transcriptome characteristics of UCB cells. **a** UMAP plots showing scRNA-seq data from immune cells and HSPCs colored by cell types (left), neonates, and groups (right). **b** UMAP plots showing scRNA-seq data from HSPCs colored by cell types (left), neonates, and groups (right). **c** Dot plot showing the expression profiles of cell type-specific marker genes among UCB cells. Dot size indicates the percentage of expressed cells, and dot color indicates the scaled expression level. For each gene, the expression level is scaled among cell types. **d** Stacked barplots showing the percentages of different cell types among immune cells (left) and HSPCs (right). **e** Dot plot showing the log_2_-transformed fold change of genes between IS and NI groups. These genes are related to mitochondrial respiratory chain complexes. Dot color indicates the log_2_-transformed fold change, and dot size indicates the absolute value of the log_2_-transformed fold change. **f** Dot plot showing biological process GO terms corresponding to upregulated DEGs in IS HSPCs compared to NI HSPCs. Dot size indicates the ratio of related genes among inputs, and dot color indicates the statistical significance. **g** Boxplot showing the gene module score of the hallmark of TNF-α signaling via NFκB among HSPCs. *P*-values from two-tailed Student’s *t*-tests are indicated. **h** Boxplot showing the gene module scores of LMPP-related hematopoiesis-related TFs. These TFs, collected from a public study, are highly expressed in LMPPs compared to GMPs and are affiliated with the myeloid lineage. *P*-values from two-tailed Student’s *t*-tests are indicated.

Unsupervised clustering and UMAP visualization revealed nine distinct cell clusters with specific expression of well-known lineage marker genes: CD34^+^ cell (*CD34*), T cell (*CD2* and *CD3D*), B cell (*CD19* and *CD79A*), CD16^+^ natural killer (NK) cell (*KLRF1*, *NKG7*, and *FCER3A*), CD56^+^ NK cell (*NCAM1*), cycling NK cell (*MKI67* and *TOP2A*), monocyte (*CD16*, *FCN1*, and *FCER3A*), and dendritic cell (DC) (*FCER1A*) (Fig. 6a; Supplementary Fig. S5c). Furthermore, we identified eight HSPC subtypes among CD34^+^ cells with specific expression of well-known lineage marker genes: hematopoietic stem cell (HSC) and multipotent progenitor (HSC/MPP) (*NRIP1* and *SELL*), lymphoid-primed MPP (LMPP) (*COBLL1* and *MME*), granulocyte-monocyte progenitor (GMP) (*AZU1* and *MPO*), megakaryocyte-erythroid progenitor (MEP) (*PBX1* and *PLEK*), basophil-eosinophil-mast (BEM) cell progenitor (*HDC* and *LMO4*), erythroid progenitor (Ery) (*GATA1* and *KLF1*), innate lymphoid cell (ILC) precursor, and plasmacytoid DC (pDC) precursor (*CCDC50*) (Fig. 6b; Supplementary Fig. S5d). We identified cell type-specific marker genes among immune cells and HSPCs, and their expression profiles supported our annotations (Fig. 6c; Supplementary Table S5).

Although adaptive and innate immune cells continue to develop to mature status after birth^44^, we identified cellular and molecular differences in immune cells between the two groups at the ‘baseline’ of the whole life. Compared to the NI group, the IS group showed a slight decrease in abundance of CD16^+^ NK cells and a slight increase in monocytes, suggesting the presence of an inflammatory environment in neonates (Fig. 6d; Supplementary Fig. S6a). This finding implied the existence of emergency myelopoiesis (EM), a process by which the hematopoietic system rapidly generates myeloid cells at the expense of other blood lineages in response to inflammation^45^, consistent with the slightly higher levels of inflammatory cytokines in UCB serum (Fig. 5h). When we identified DEGs between the two groups, we observed more DEGs in monocytes among immune cells, reflecting transcriptomic differences in monocytes between the IS and NI groups (Supplementary Fig. S6b). GO analysis revealed that downregulated DEGs were enriched in genes related to OXPHOS (e.g., *COX2*, *COX8A*, *ATP5F1E*, *NDUFA4*, and *UQCRB*) (Supplementary Fig. S6c). Specifically, for CD16^+^ NK cells and monocytes, upregulated DEGs were enriched in positive regulation of immune response (e.g., *IFI6*, *HLA-B*, *FOXP1*, and *TLR10*), activation of innate immune response (e.g., *CREBBP* and *BTK*), and chemotaxis (e.g., *ANXA1*, *S100A8*, *CSF3R*, *CX3CR1*, and *IL6R*) (Supplementary Fig. S6c). These results indicated activated immune responses in IS neonates, suggesting that exposure to maternal acute SARS-CoV-2 infection may imprint the immune cells in the offspring. Interestingly, when we examined the expression level of hallmark genes related to TNF-α signaling via NFκB, a classic immune response pathway, we found that they were downregulated in all immune cells except monocytes (Supplementary Fig. S6d). This supports the activation of innate immune responses in monocytes from IS neonates.

### Lower abundance of HSCs/MPPs, inhibited OXPHOS-related genes, and redirection of LMPPs toward the myeloid lineage in the IS group

We identified DEGs between the two groups in HSPC subtypes and revealed 1560 downregulated and 1119 upregulated DEGs in IS neonates, suggesting dramatic transcriptomic differences between the two groups (Supplementary Fig. S6b and Table S5). Similar to downregulated DEGs in immune cells, downregulated DEGs in HSPCs, including *NDUFA1/2/3/4*, *SDHA*, *UQCR10/11*, *ND1/2/3/4/4* *L/5*, *COX1/2/3*, and *ATP5F1E*, were also enriched in OXPHOS, electron transport chain, and ATP metabolic process (Fig. 6e; Supplementary Fig. S6e). We noted that genes related to mitochondrial respiratory chain complexes I–V were commonly downregulated in HSPCs from the IS group, indicating decreased mitochondrial activation (Fig. 6e). Since the primary energy source shifts from glycolysis to OXPHOS during HSC differentiation^46–48^, we inferred that reduced mitochondrial activation, along with lower levels of reactive oxygen species (ROS) byproducts which impair HSC function, may contribute to HSC protection in neonates exposed to an inflammatory environment. The upregulated DEGs were associated with regulation of mitotic cell cycle (e.g., *ACTB* and *CDKN1A*), antigen processing and presentation of peptide antigen (e.g., *CD74*, *HLA-B*, and *HLA-DRA*), and regulation of immune effector process (e.g., *B2M* and *JUNB*) (Fig. 6f). Furthermore, among HSPC subtypes in the IS group, we detected a commonly increased expression of hallmark genes related to TNF-α signaling via NFκB, suggesting that IS HSPCs were imprinted with the maternal acute infection event and may have a long-lasting effect on offspring health (Fig. 6g). Notably, genes related to the regulation of myeloid cell differentiation (e.g., *FOS*, *NFKBIA*, *GATA2*, and *STAT1*) and cell fate determination (e.g., *MEF2C*, *MCL1*, and *KLF4*) were upregulated in the IS group (Fig. 6f).

Among HSPCs, the abundance of HSCs/MPPs was significantly lower (one-tailed Wilcoxon rank sum test, *P* = 0.03), whereas the abundance of LMPPs was significantly higher (*P* = 0.01) in IS neonates compared to NI neonates (Fig. 6d; Supplementary Fig. S6a). By contrast, the abundance of GMPs, which differentiate into monocytes and granulocytes, was slightly lower in the IS group, contradicting the slight increase in monocyte proportion (Supplementary Fig. S6a). In adults, EM functions at multiple levels of the HSPC hierarchy, all geared toward rapid amplification of myeloid cell production at the expense of other blood lineages, followed by the restoration of homeostatic levels^45,49^. EM activation redirects lymphoid-biased progenitors toward the myeloid lineage^50^ and overproduces myeloid-biased progenitors^51^. We therefore hypothesized that the pro-inflammatory environment in IS neonates induced EM and redirected LMPPs toward the myeloid lineage. To test this hypothesis, we examined the expression of monocyte-specific marker genes in all HSPCs. These genes were highly expressed in GMPs, as expected, and interestingly, they exhibited significantly higher expression levels in IS LMPPs compared to NI LMPPs (Supplementary Fig. S6f). We also collected hematopoiesis-related TFs that were highly expressed in LMPPs compared to GMPs and these TFs were affiliated with the myeloid lineage from the previous study^52^. Expression levels of these TFs were significantly higher in IS LMPPs than in NI LMPPs (Fig. 6h), and this was accompanied by upregulation of genes involved in myeloid cell differentiation (Fig. 6f). These results supported the redirection of LMPPs into myeloid lineages in neonates derived from mothers with acute SARS-CoV-2 infection.

### Association between altered HSPC differentiation bias and TE-derived placentas

Hematopoietic progenitor development originates from the mesoderm derived from ICM cells. However, our analysis focused primarily on TE cells, with limited ICM representation due to technical constraints on preimplantation embryo sampling. We acknowledged the temporal disconnect between TE-specific changes in gene expression and neonatal hematopoietic phenotypes. To establish a potential link between TE-specific transcriptional changes and neonatal myeloid bias of HSPCs, we next conducted integrated multi-tissue analyses.

**Fig. 7:**
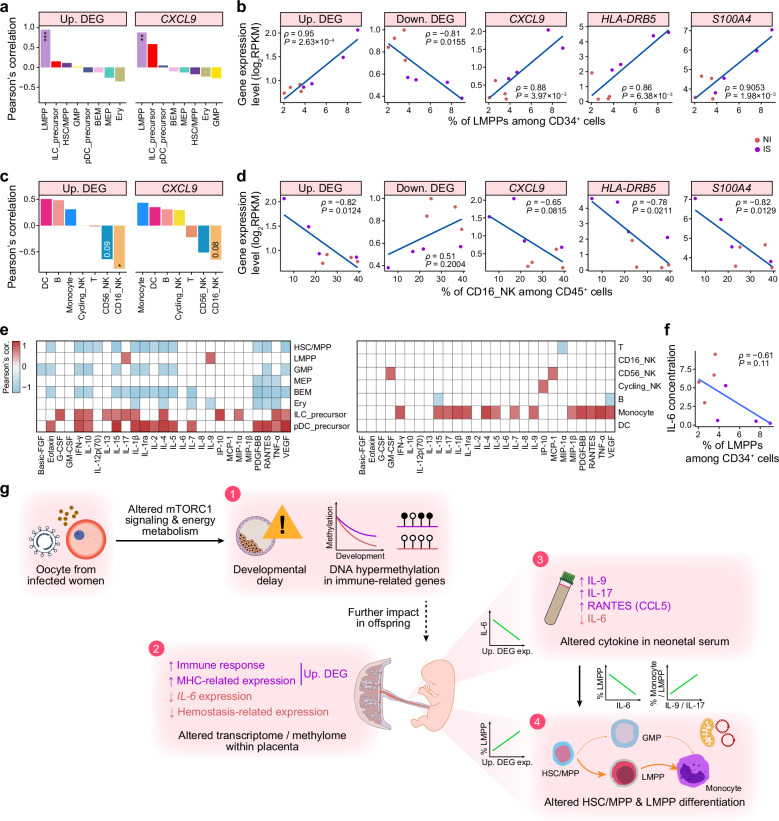
Correlation between the pro-inflammatory response within the placenta and the abundance of distinct CD34^+^/CD45^+^ cell types among UCB cells. **a** Barplots showing Pearson’s correlation coefficients between the abundance of different CD34^+^ cell types and the expression of (all or representative) upregulated/downregulated DEGs in IS placentas. ***P < 0.001, **P < 0.01, and *P < 0.05 (significance of Pearson’s correlation). **b** Scatter plots showing Pearson’s correlations between the expression of (all or representative) upregulated/downregulated DEGs in IS placentas and the abundance of LMPPs in UCB cells. Pearson’s correlation coefficients (*ρ*) and the significance of correlation tests (*P*) are indicated. **c** Barplots showing Pearson’s correlation coefficients between the abundance of different CD45^+^ cell types and the expression of (all or representative) upregulated/downregulated DEGs in IS placentas. *P < 0.05 (significance of Pearson’s correlation). **d** Scatter plots showing Pearson’s correlation coefficients between the expression of (all or representative) upregulated/downregulated DEGs in IS placentas and the abundance of CD16^+^ NK cells in UCB cells. Pearson’s correlation coefficients (*ρ*) and the significance of correlation tests (*P*) are indicated. **e** Heatmaps showing Pearson’s correlation coefficients between the abundance of different CD34^+^ (left)/CD45^+^ (right) cell types and cytokine concentrations. Only correlation coefficients with *P* ≤ 0.1 are displayed. **f** Scatter plot showing the Pearson’s correlation coefficient between LMPP abundance and IL-6 concentration. Pearson’s correlation coefficient (*ρ*) and the significance of the correlation test (*P*) are indicated. **g** Schematic diagram illustrating that in the IS group, (1) dysregulated mTORC1 signaling and aberrant energy metabolism contribute to blastocyst developmental delay, characterized by abnormal methylation patterns of immune-related genes. Moreover, (2) activation of placental inflammation, (3) increased IL-9/IL-17 levels and decreased IL-6 levels in UCB serum, and (4) downregulation of OXPHOS-related genes and a skewed differentiation bias of LMPPs to support EM within HSPCs derived from UCB cells are detected in the IS group.

We first assessed whether placental gene expression profiles were correlated with neonatal HSPC composition, and we conducted Pearson’s correlation analysis between placental bulk RNA-seq data and UCB cellular abundances. Notably, among CD34^+^ HSPCs, the LMPP was the only cell type whose abundance was strongly correlated with the average expression level of upregulated DEGs identified in IS placentas (Pearson’s correlation coefficient *ρ* = 0.95, correlation test, *P* = 2.63 × 10^–4^) (Fig. 7a, b). This relationship was further supported by significant positive correlations between LMPP abundance and the expression of individual upregulated DEGs, including *CXCL9*, *S100A4*, and *HLA-DRB5* (Fig. 7a, b; Supplementary Fig. S7a). By contrast, the average expression level of downregulated DEGs in IS placentas was negatively correlated with LMPP abundance (*ρ* = −0.81, *P* = 0.02) (Fig. 7a, b). Intriguingly, we detected a significant negative correlation between placental upregulated DEG expression and NK cell abundance, particularly for CD16^+^ NK cells (*ρ* = −0.82, *P* = 0.01) (Fig. 7c, d; Supplementary Fig. S7b). These findings indicated that placental pro-inflammatory activation was associated with an expansion of LMPPs but a reduction in NK cells within UCB, highlighting a potential mechanistic link between placental inflammation and fetal hematopoietic reprogramming.

In addition to placental pro-inflammatory responses, we also examined the relationships between UCB serum cytokine concentrations and hematopoietic cell populations. Levels of most cytokines were negatively correlated with CD34^+^ HSPC abundance, with the notable exception of LMPPs, ILC precursors, and pDC precursors (Fig. 7e, left). By contrast, multiple cytokines demonstrated positive correlations with monocyte abundance (Fig. 7e, right). Notably, we observed a negative relationship between LMPP abundance and UCB serum IL-6 levels, consistent with previous literature demonstrating the critical role of IL-6 in promoting myelopoiesis in vitro and in vivo^53–55^ (Fig. 7f). These results suggest that the UCB inflammatory milieu is related to the cellular composition of HSPCs and monocyte production, consistent with EM.

On the basis of these observations, we propose that both placental inflammation and fetal cytokine responses (particularly IL-6, IL-9, and IL-17) may synergistically promote myeloid-biased differentiation of LMPPs (Fig. 7g). This mechanism is consistent with a previous report that placental inflammation can disrupt fetal neurological development through brain cytokine balance^56^. Notably, maternal SARS-CoV-2 infection during pregnancy has been associated with both placental inflammation and neurodevelopmental abnormalities in offspring^56^, suggesting potential mechanisms similar to our findings.

## Discussion

Due to the scarcity of human samples, it has been challenging to determine the short- and long-term impacts of maternal acute environmental exposure. Our study investigated the impact of maternal acute SARS-CoV-2 infection on embryo development and offspring health at the molecular level using human preimplantation embryos and UCB. Our findings provide insight into the mechanisms by which embryo development is affected (Fig. 7g).

Although worse embryo outcomes associated with SARS-CoV-2 infection have been reported previously^9,10^, the exact mechanisms of this effect remain unclear. Since SARS-CoV-2 RNA is not detected in ovarian tissue, follicular fluid, cumulus cells, endometrial tissue, or vaginal fluid samples^4–6^, this phenotype may not be caused by the direct effect of viral oocyte invasion. Our scRNA-seq analysis demonstrated a reduction in the expression of OXPHOS-related genes and an increase in the expression of glycolysis-related genes within IS blastocysts. This finding mirrors the inhibition of mitochondrial function in blastocysts derived from oocytes retrieved from SARS-CoV-2-positive women. SARS-CoV-2 viral proteins have been reported to bind to host mitochondrial proteins to inhibit OXPHOS, increase ROS levels, and shift metabolism toward glycolysis to drive viral biogenesis in humans or mice^57,58^. In addition, mitochondrial genes were downregulated in the cerebellum of hamsters during SARS-CoV-2 infection, even though no SARS-CoV-2 was detected in the brain^58^. This may be caused by a systemic host response, followed by viral suppression of mitochondrial gene transcription and induction of glycolysis, leading to the deployment of antiviral immune defenses^58^. Moreover, impaired mitochondrial function persisted in the heart, kidney, liver, and lymph nodes after virus clearance^58^. Therefore, mitochondrial function may also be impaired in the female reproductive system. Furthermore, ROS induced by SARS-CoV-2 infection^59^ can affect multiple aspects of reproductive physiology, including oocyte maturation, fertilization, and embryo development^60^. Aberrant systemic inflammation related to high levels of pro-inflammatory cytokines^61^ may be another possible cause.

We observed the downregulation of mTORC1 signaling in IS blastocysts. The mTOR pathway regulates many cellular processes in female reproduction, including folliculogenesis^62^, oocyte meiotic maturation^63^, and the response of the embryo to intracellular and extracellular nutrition^64,65^. Notably, mTOR signaling controls embryonic diapause^66^ and early post-implantation developmental failure^27,67^. Although the nutritional and metabolic requirements during preimplantation development are minimal, some primary nutrients or microenvironmental cues are essential for the zygotic transition and the morula-stage transition, two key early preimplantation events in the initiation of ZGA and cell fate commitment^68,69^. Therefore, dysregulated mTORC1 signaling involving nutritional deficiencies or excesses, as well as suppressed mitochondrial function, compromises embryo quality and leads to delayed blastocyst formation in the IS group.

Our study revealed a trend toward increased inflammatory cytokine concentrations in IS UCB serum, and consistent with this finding, we detected an increase in monocyte abundance, reflecting the activation of myelopoiesis in response to inflammation, a process known as EM. We also observed that the abundance of LMPPs, rather than GMPs, was significantly higher in IS neonates, and the abundance of HSCs/MPPs was lower, reflecting a redirection of LMPP differentiation bias toward the myeloid lineage. Notably, *SPI1* (PU.1), a pioneer TF with a critical role in myelopoiesis^70^, was not detected in LMPPs, but downstream PU.1 target genes^71^ were upregulated in the IS group. These included the granulocyte colony-stimulating factor (G-CSF) receptor *CSF3R* in monocytes and AP-1 TFs (e.g., *JUN*, *JUNB*, *JUND*, *FOS*, and *FOSB*) in LMPPs (Supplementary Table S5). Therefore, the detailed mechanism driving EM remains to be explored.

We observed an unexpected upregulation of immune response-related genes in both HSPCs and immune cells in the IS group. This phenomenon may be associated with abnormal DNA methylation patterns of immune-related genes in IS blastocysts and immune activation in IS placentas, suggesting that maternal acute infection exposure may epigenetically imprint offspring and potentially exert long-lasting effects on offspring health.

Interestingly, we identified a consistent downregulation of OXPHOS-related genes in both blastocysts and UCB cells from the IS group. Mitochondria generate ROS as byproducts of oxidative respiration, and HSCs are sensitive to cellular redox states, which is why they preferentially localize in bone marrow niches characterized by low ROS levels^72^. Maintenance of ROS homeostasis is crucial for HSC function, as excessive ROS levels can impair HSC functionality, and subtle fluctuations may play a regulatory role in determining HSC fate^73^. Current understanding suggests that HSCs maintain their quiescent state through restricted mitochondrial metabolism, thereby maintaining low ROS levels and preventing premature commitment and differentiation^74^. By contrast, HSC differentiation and lineage commitment are associated with elevated ROS levels, activation of mTOR signaling, and enhanced mitochondrial biogenesis^75,76^. On the basis of these observations, we propose that the observed inhibition of OXPHOS-related genes may represent a protective mechanism for HSCs, potentially preventing premature commitment and differentiation through maintenance of appropriate ROS levels in an inflammatory environment.

In conclusion, our study demonstrates that oocyte retrieval in the presence of SARS-CoV-2 infection can have harmful effects on embryo development and reveals potential mechanisms underlying impaired blastocyst formation. Furthermore, we reveal that maternal acute SARS-CoV-2 infection has a long-lasting impact on the hematopoietic system of early offspring. Our study calls attention to the need for further research to achieve the best balance between patient safety and other interests in ART.

### Limitations

Several limitations should be considered in this study. First, the relatively small sample size of preimplantation embryos may introduce potential bias in cell type representation and sex distribution. Specifically, the low capture efficiency of ICM cells, along with the absence of sufficient male embryos in our analysis, could potentially affect our conclusions. Second, the absence of maternal peripheral cytokine profiling may constrain our ability to establish a direct causal relationship between maternal infection and fetal hematopoietic changes. Third, the absence of oocytes from SARS-CoV-2-positive women prevented us from determining the cause of inhibited mitochondrial function and mTOR signaling in blastocysts from the IS group. Finally, post-pandemic ethical regulations protecting patient privacy currently prohibit direct inquiries about SARS-CoV-2 infection status, preventing us from expanding our cohort through additional sample collection (including oocytes and embryos).

## Materials and methods

### Ethics

This study was approved by the Institutional Ethics Committee of the International Peace Maternity and Child Health Hospital, School of Medicine, Shanghai Jiao Tong University (Approval# GKLW-2021-18).

### Statistical note about the clinical information

Although both the IS and NI groups demonstrated identical median cleavage rates of 100% (IQR: 100%–100%) across all samples, the IS group showed statistically significantly lower cleavage rates overall (two-tailed Wilcoxon rank sum test, *P* = 0.043). This apparent discrepancy can be explained by examining the distribution tails: at the 10th percentile, cleavage rates were markedly reduced in the IS group (73.33%) compared to the NI group (100%). This finding suggested that although most samples in both groups achieved optimal cleavage, a subset of IS samples exhibited compromised performance, resulting in the observed statistical difference.

### Statistical adjustment for the potentially confounding factors of age and embryo identity

We used a linear regression model implemented with the R function *lm* to account for age-related effects when analyzing clinical parameters. For example, when evaluating the rate of blastocysts from good-quality day 3 embryos, we constructed the following model: rate ~ disease_group + age.

In comparative analyses of key pathway expression levels between NI and IS blastocysts, we incorporated embryo identity as a confounding factor using the model: pathway_expression ~ disease_group + embryo.

### Collection of early human embryos and single-cell isolation

All embryos used in this study were collected from the Reproductive Medicine Center of the International Peace Maternity and Child Health Hospital, School of Medicine, Shanghai Jiao Tong University. The donated blastocysts were cryopreserved via vitrification at the reproductive center, with each blastocyst individually frozen on a cryostorage carrier. Commercial vitrification and warming solution kits (Kitazato Corporation, Japan) were used for blastocyst cryopreservation and thawing procedures. After thawing, the blastocysts were cultured in vitro using G2 Plus medium (Vitrolife, Sweden) for 2 h (37 °C, 6% CO_2_, 5% O_2_). The zona pellucida was subsequently removed through gentle pipetting with acidic Tyrode’s solution (Sigma), followed by three washes in phosphate-buffered saline (PBS) containing 0.5% bovine serum albumin (BSA). Laser-assisted ablation (RI Scientific Instruments) was used to release blastocoelic fluid from zona-free blastocysts, which were then transferred to a digestion medium (accutase medium: 0.25% trypsin, 1:1) and incubated at 37 °C for 30–40 min. To facilitate physical dissociation into individual cells, the digested blastocysts were subjected to gentle mechanical pipetting using sequentially smaller-bore glass pipettes (varying inner diameters). Following complete dissociation, isolated single cells were washed twice in PBS supplemented with 0.1% BSA. Finally, individual cells were transferred to lysis medium on ice using a mouth-controlled micropipette. All procedures were performed under a stereomicroscope.

### UCB collection and nucleated cell isolation

UCB samples were collected from eight neonates, including four neonates derived from oocytes collected from mothers with acute SARS-CoV-2 infection and four neonates from mothers without a history of SARS-CoV-2 infection. Approximately 10 mL of UCB was collected from each donor using EDTA-containing anticoagulant tubes, and all samples were transported to the laboratory within 60 min post-collection. Written informed consent was obtained from all participants or their legally authorized representatives prior to sample collection.

UCB samples were processed using Ficoll density gradient centrifugation to isolate mononuclear cells. In brief, anticoagulated whole blood was carefully layered onto human peripheral blood lymphocyte separation medium (TBD, LTS1077). The samples were centrifuged at 700× *g* for 25 min at 25 °C. Following centrifugation, the mononuclear cell layer at the plasma-separation medium interface was collected and diluted with PBS, then centrifuged at 250× *g* for 10 min. The supernatant was discarded, and residual red blood cells were lysed using erythrocyte lysis buffer (Solarbio, R1010) with incubation on ice for 10 min. Cells were washed with wash buffer (PBS containing 0.04% BSA) and centrifuged at 300× *g* for 10 min at 4 °C. The cell pellet was resuspended, and cells were counted using a fluorescence cell counter. For long-term storage, cells were resuspended in freezing medium, aliquoted into cryovials, and transferred to liquid nitrogen after gradient freezing.

### Construction of scRNA-seq libraries for blastocysts

The scRNA-seq libraries were prepared using a modified STRT-seq method^21,77,78^. Individual cells were isolated using a mouth pipette and transferred into the lysis buffer. mRNA from the lysate was reverse transcribed using SuperScript II Reverse Transcriptase (Thermo Fisher Scientific, 18064022), and cDNA from each cell was tagged with a unique 8-nucleotide (nt) cell-specific barcode. The second cDNA strand was then synthesized, and the cDNA was pre-amplified. The amplified cDNA products were pooled, purified, and fragmented. Library preparation was performed using the KAPA HyperPrep Kit (KAPA, KK8504), and sequencing was carried out on the Illumina HiSeq 4000 platform (Novogene) to obtain 150-bp paired-end reads.

### Construction of PBAT DNA methylome sequencing libraries for blastocysts

The single-cell whole-genome bisulfite sequencing procedure followed the established PBAT method^38^. In brief, individual cells were transferred to the lysis buffer by mouth pipetting. Genomic DNA was isolated through proteinase K digestion at 50 °C, followed by bisulfite conversion using the EZ DNA Methylation Kit (Zymo Research, D5044) according to the manufacturer’s protocol. The DNA was supplemented with the biotinylated random primer Bio-P5-N9 and 50 units of Klenow polymerase (3′ to 5′ exo-, New England BioLabs). This random priming process was performed five times. Second strands were then synthesized using a different random primer, P7-N9, and the final libraries were created after 8–12 cycles of PCR amplification using the Illumina universal PCR primer and Illumina indexed primer. Finally, the constructed library was sequenced on the Illumina HiSeq 4000 platform (Novogene) to obtain 150-bp paired-end reads.

### Construction of bulk RNA-seq libraries for placental tissues

For bulk RNA sequencing, total RNA was isolated from placental tissues with an RNA Extraction Kit (Vazyme, RC113-01), and library construction was performed using the VAHTS Universal V10 RNA-seq Library Prep Kit (Vazyme, NR611).

### Construction of WGBS libraries for placental tissues

Genomic DNA was extracted from placental tissues using the DNA Extraction Kit (TIANGEN Biotech, DP705). For library preparation, 2 μg of genomic DNA was fragmented to ~300 bp using the ME220 Focused Ultrasonicator (Covaris, 500217). Fragmented DNA underwent end repair, 3′-end adenylation (A-tailing), and ligation with methylation adapters (New England BioLabs, E7140S). Bisulfite conversion was performed using the DNA Methylation-Direct MagPrep Kit (Zymo Research, D5044) under optimized conditions: denaturation at 98 °C for 10 min, followed by conversion at 64 °C for 3.5 h. Final libraries were constructed through 4–6 cycles of PCR amplification with KAPA HiFi Uracil+ DNA Polymerase (Roche, KK2801) using Illumina universal and indexed primers, followed by size selection using AMPure XP beads (Beckman Coulter, A63881).

### Isolation of human HSPCs from neonatal UCB samples

For HSPC isolation, the nucleated cells were first stained with BD Horizon Fixable Viability Stain 660 (BD Biosciences, 564405) for 15 min at room temperature in the dark, and the staining reaction was terminated by the addition of fetal bovine serum. The cells were then stained with PE-Cy7-conjugated mouse anti-human CD34 antibody (BD Biosciences, 564405) for 20 min under the same conditions, followed by termination with fetal bovine serum. The cells were resuspended in cold RPMI-1640 medium supplemented with 10% FBS, and hCD34^+^ cells were sorted using a flow cytometer (BD FACSAria) for subsequent 10× Genomics sequencing.

### Microfluidic droplet single-cell analysis (10× Genomics)

Single cells were encapsulated in droplet emulsions using the 10× Genomics Chromium Single Cell Instrument according to the manufacturer’s protocol, with scRNA-seq libraries constructed using the 10× Genomics Chromium Single Cell 3′ GEM Library & Gel Bead Kit v2. In brief, cells were loaded into each channel with a target recovery of ~5000 cells per sample, and cell concentration was measured using the Moxi GO II flow cytometer (Orflo Technologies) to ensure optimal loading density. All reactions were performed on a Bio-Rad C1000 Touch Thermal Cycler with a 96-deep-well reaction module, and cDNA amplification and sample indexing were carried out using 12 cycles. The amplified cDNA and final libraries were assessed using an AATI Fragment Analyzer with the High Sensitivity NGS Kit (Advanced Analytical). The average fragment length of the 10× cDNA libraries was evaluated using the AATI fragment analyzer, and library quantification was performed by qPCR using the KAPA Library Quantification Kit. All libraries were diluted to a final concentration of 2 nM and pooled for each sequencing run on the Illumina NovaSeq 6000 platform.

### Cytokine profiling in neonatal UCB serum

Serum samples were isolated from UCB and subjected to cytokine analysis using a customized Luminex Human Cytokine 27-plex panel (LabEx, LX-MutiDTH-27). The panel included the following analytes: FGF basic, Eotaxin, G-CSF, GM-CSF, IFN-γ, IL-1β, IL-1rα, IL-2, IL-4, IL-5, IL-6, IL-7, IL-8, IL-9, IL-10, IL-12, IL-13, IL-15, IL-17, IP-10, MCP-1, MIP-1α, MIP-1β, PDGF-BB, RANTES, TNF-α, and VEGF. Cytokine quantification was performed on 60 μL of serum following the manufacturer’s protocol.

### Processing of scRNA-seq data from blastocysts

The initial processing of raw single-cell RNA-seq data involved the removal of template switch oligo (TSO) and poly(A) tail sequences. Reads containing adapters and reads with low-quality bases were filtered out to obtain clean reads. The clean reads were aligned to the human reference genome (hg38) using *TopHat* software (version 2.0.12)^79^. To ensure accuracy, only reads with unique mapping based on UMI counts were considered, and reads sharing the same UMI sequence were counted as a single entity using *HTSeq* software^80^. Gene expression levels were quantified as transcripts per million (TPM). Specifically, the TPM value for gene *g* in cell *c* (TPM_*g,c*_) was calculated by dividing the UMI count of gene *g* by the total UMI counts in cell *c* and then multiplying by 1,000,000. Given that the complexity of scRNA-seq libraries was estimated at ~100,000 transcripts, gene expression levels were normalized using the transformation log_2_(TPM/10 + 1).

To obtain high-quality single cells, cells were subjected to stringent filtering criteria: (1) detection of at least 1000 genes, and (2) a maximum of 1,500,000 detected UMIs. After this filtering process, 224 high-quality single cells from blastocysts were retained for further analysis.

### Identification of cell types and DEGs among clusters in blastocysts

The scRNA-seq data, quantified with raw UMI counts, were imported into the R package *Seurat*^81^ (version 5.0.2) to identify cell types in the blastocysts. Normalization of UMI counts was performed using the *NormalizeData* function in *Seurat* with default parameters. The data were then scaled using the *ScaleData* function, incorporating all detected genes. The *FindVariableFeatures* function was used with default parameters to identify highly variable genes (HVGs), and PCA was then performed using the *RunPCA* function with the selected HVGs. The first 20 PCA dimensions were used for non-linear dimensionality reduction analysis via UMAP with the *RunUMAP* function. Unsupervised clustering analysis was performed to categorize all single cells into five distinct clusters using the *FindClusters* function with the clustering ‘*resolution*’ parameter set to 0.2. The Wilcoxon rank sum test was used to detect DEGs among clusters with the *FindAllMarkers* function. Genes were classified as DEGs if they met the following criteria: (1) a log_2_-transformed fold change greater than 1, (2) a *P*-value below 0.05, and (3) expression in > 25% of cells in the corresponding cluster.

### Calculation of cell cycle status

For the cell cycle analysis, we used a gene set comprising 43 G1/S-phase genes and 54 G2/M-phase genes, as described in previous studies^82,83^. The cell cycle status of each cell was inferred on the basis of the average expression levels of these two gene sets. Cells were classified as quiescent if the expression levels of both G1/S and G2/M genes were below 1; otherwise, they were categorized as proliferative^84^. Among proliferative cells, those with higher expression of G1/S genes than G2/M genes were assigned to the G1/S phase, and those with higher expression of G2/M genes were assigned to the G2/M phase. For cells in the G1/S phase, if the expression of G2/M genes exceeded 1, they were classified as being in the S phase; otherwise, they were classified as being in the G1 phase.

### Establishment of cell-scoring models to infer developmental delay using machine learning

To analyze developmental delays in blastocysts, we established a cell-scoring model for individual cells using well-defined scRNA-seq data from a previous study^22,23^, similar to a previous report^85^. We first calculated the pattern distribution of well-defined cells^22,23^. Then, using this reference distribution, we compared our single blastocyst cells from the IS and NI groups.

We first trained the cell-scoring model using public scRNA-seq data from oocytes and different-stage embryos^22^. Gene expression levels from these data were quantified as RPKM (reads per kilobase per million mapped reads). To avoid the effect of housekeeping genes, we selected the top-ranked 3000 HVGs (based on CV-mean values) to train the cell-scoring model. A logistic regression model with L2-norm regularization and a multinomial learning approach was then trained using the log_2_-transformed max-normalized data with the *LogisticRegression* function in the Python package *scikit-learn*^86^. The trained model was used as a reference to predict the probabilities of our single cells, and our scRNA-seq data were quantified as TPM. The predicted probabilities were calculated using the *softmax* function.

We then established a cell-scoring model based on another set of scRNA-seq data from preimplantation embryos collected at different developmental days^23^. The UMI count data from these public data were used to train the model, and the UMI count data from our scRNA-seq were used to calculate the probabilities.

### Identification of DEGs in TE cells between the IS and NI groups

To identify DEGs in TE cells between the two groups, we analyzed the scRNA-seq data quantified as log_2_-transformed TPM, with the Wilcoxon rank sum test using the *FindAllMarkers* function in *Seurat*. DEGs were defined as genes that met the following criteria: (1) a log_2_-transformed fold change greater than 1, (2) a *P*-value below 0.05, and (3) expression in more than 25% of cells in the respective group. Notably, we first identified DEGs by including scRNA-seq data from NI_Embryo8 (male), but the expression profiles of DEGs in cells from NI_Embryo8 differed from those in other cells from the NI group. Therefore, we ultimately excluded TE cells from NI_Embryo8 and re-identified DEGs between the two groups. GO analysis was performed using the web tool *Metascape* with default settings.

### Identification of ZGA-related genes and calculation of corresponding expression levels

We identified ZGA-related genes on the basis of RPKM-quantified scRNA-seq data from a previous study^22^. ZGA-related genes were defined using the following criteria: (1) no expression before the 8-cell stage: the average expression level in all previous stages (MII-oocyte, zygote, 2-cell, or 4-cell) was less than 1, indicating minimal or no expression at these four stages; (2) significant upregulation at the 8-cell stage: the average expression level in 8-cell embryos was at least twice that in 4-cell embryos (*P*-value ≤ 0.01, determined by the Wilcoxon rank sum test implemented with the *FindAllMarkers* function in *Seurat*); (3) expression at the 8-cell stage: the average expression level in 8-cell embryos exceeded 1; and (4) overlap with scRNA-seq data from blastocysts in this study. Using these criteria, we obtained 865 ZGA-related genes.

After the identification of ZGA-related genes, we computed their mean expression levels at each embryonic stage. These genes were clustered into five subgroups using the R package *cummeRbund* (version 2.44.0). For each subgroup, we applied the following criteria: if the mean expression decreased in either the morula or blastocyst relative to the 8-cell stage, the subgroup was classified as T-ZGA; if its expression continued to increase through the morula and blastocyst stages, it was designated as I-ZGA.

To analyze the overlap between ZGA-related genes and DEGs (TE cells between the IS and NI groups), we used the R package *GeneOverlap* (version 1.42.0) and performed Fisher’s exact test.

### Processing of single-cell DNA methylation data from blastocysts

For PBAT DNA methylation sequencing data, raw reads were initially processed to eliminate adapter sequences and low-quality bases using *trim_galore* software (version 0.3.3) with the following parameters: ‘*--quality 20, --phred33, --length 50, --stringency 3, --paired*’. Quality-filtered reads were then aligned to the human reference genome (hg38) using *Bismark* software^87^ (version 0.22.3) in paired-end mode with the parameter ‘*--non-directional*’. To maximize read recovery, unmapped reads were re-aligned using *Bismark* in single-end mode. Only uniquely mapped reads were retained for downstream analysis.

After alignment, duplicate reads were removed using the *rmdup* command in *SAMtools* software^88^ (version 1.20). For each cytosine site in the reference genome (or its corresponding guanine on the opposite strand), the DNA methylation level was calculated as the ratio of reads supporting methylated cytosines (C) to the total reads (methylated and unmethylated). The reads were extracted using the extract command in *MethylDackel* software (version 0.3.0). In this study, unless otherwise specified, the DNA methylation level of a sample or region refers specifically to the methylation level of CpG sites within that sample or region. For single-cell DNA methylome analysis, CpG sites with methylation levels exceeding 90% were classified as methylated (methylation level quantified as 100%), whereas those below 10% were considered unmethylated (methylation level was quantified as 0%)^89,90^. The minimum read coverage required to determine methylation levels was set to 1× for single cells.

### Collection of genomic elements and calculation of methylation levels

DNA methylation levels were assessed across various genomic regions, with only regions that contained at least three CpG sites included in the analysis. The methylation levels for these genomic elements were determined by averaging the methylation levels of all covered CpG sites within each region.

The whole human genome was divided into 1-kb bins. Gene bodies were defined as the regions spanning from the transcription start site (TSS) to the transcription end site (TES). To analyze DNA methylation levels around gene bodies (Supplementary Fig. S3a), the gene-body region was extended by 2 kb upstream of the TSS and 2 kb downstream of the TES. Each gene body was divided into 100 bins of various sizes, and the extended regions were split into 10 bins of 200 bp each.

Promoters were defined as regions spanning 1-kb upstream to 0.5-kb downstream of the TSS. Promoters were categorized into high-density CpG promoters (HCPs), intermediate-density CpG promoters (ICPs), and low-density CpG promoters (LCPs) on the basis of their CpG density, as defined previously^91,92^. Enhancer annotations were sourced from a previous study^93^. Genomic elements, including exons, introns, and CpG islands (CGIs), were obtained from the UCSC Genome Browser (hg38). Repetitive elements and their subfamilies, including LINE, SINE, LTR, L1, L2, Alu, MIR, ERV1, ERVK, ERVL, and ERVL-MaLR, were extracted from the ‘*RepeatMasker*’ track on the UCSC Genome Browser (hg38). Intragenic regions were classified as gene-body regions, and the remaining genomic regions were considered intergenic regions.

The genomic coordinates of human ICRs were obtained from a previous study^39^. To ensure compatibility with current genomic annotations, we converted these coordinates from the hg19 assembly to the hg38 assembly using the offline software *liftOver* (UCSC toolkits).

### PCA of preimplantation embryo DNA methylomes

Prior to dimensionality reduction, we preprocessed the single-cell DNA methylation data by: (1) removing genomic regions that contained exclusively missing values (*NA*) across all cells, and (2) performing an initial PCA using probabilistic PCA (*method* = *‘ppca’*) to handle missing values using the *pca* function with *completeObs* imputation in the *pcaMethods* R package (version 1.94.0). We then performed PCA on imputed methylation profiles using the *prcomp* function.

To infer the developmental stage of blastocysts from this study, we integrated a publicly available single-cell DNA methylation dataset from human preimplantation embryos, including metaphase II oocytes (*n* = 35 cells), sperm (*n* = 20), 8-cell embryos (*n* = 48), morula (*n* = 58), ICM (*n* = 108), and TE (*n* = 96)^38^. The public DNA methylation data were also generated using the PBAT method. Genomic coordinates were harmonized to the hg38 genome version using the offline software *liftOver* (UCSC toolkits) prior to analysis.

We performed PCA by combining our blastocyst data with the public dataset using the PCA method described above. Developmental relationships were quantified by computing Euclidean distances within the PCA space between blastocysts from the NI/IS groups and publicly available human preimplantation embryos using the *dist* function from the R package *proxy* (version 0.4.27).

### Identification of DMRs and DMPs using human preimplantation embryos

DMRs and DMPs in blastocysts were identified between the two groups using CpG sites with at least 1× coverage. For DMR identification, the whole genome was segmented into 300-bp fixed windows. Windows that contained at least one CpG site were retained for further analysis. DMRs between the two groups were identified using the following criteria: (1) an average methylation difference exceeding 60%, and (2) a two-tailed Student’s *t*-test *P*-value ≤ 0.01. To identify DMPs, promoters that contained at least one CpG site were retained for further analysis. DMPs between the two groups were identified using the following criteria: (1) an average methylation difference exceeding 20%, and (2) a two-tailed Student’s *t*-test *P*-value ≤ 0.05. Notably, for a given candidate region, all CpG sites in all single cells from a given group were used to calculate the average methylation level and perform statistical tests.

Using publicly available DNA methylation data from human preimplantation embryos^38^, we also identified DMRs (between 8-cell embryos and TE cells, and between morula and TE cells) and DMPs (between morula and TE cells) using the same analytical parameters described above. For comparative analysis, we assessed the overlap between our experimentally derived DMRs and those identified from the public data using the *intersect* subcommand from *bedtools* software (version 2.31.1). Only regions that showed at least 10% reciprocal overlap were considered overlapping regions.

### Processing of bulk RNA-seq data from placentas and identification of DEGs

After standard quality control steps, raw bulk RNA-seq data were processed through our customized scripts. Initial preprocessing included the removal of adapter-contaminated sequences and low-quality reads to generate high-quality clean reads for downstream analysis. These processed reads were aligned to the UCSC human reference genome (hg38) using *TopHat* software. After alignment, uniquely mapped reads were quantified using *HTSeq* software. Gene expression was quantified using RPKM normalization to account for variations in gene length and sequencing depth.

To identify DEGs between placentas from the IS and NI groups, we used the R package *DESeq2* (version 1.42.1)^94^, implementing the Benjamini–Hochberg procedure to control the false discovery rate (FDR). DEGs were defined as genes that met the following criteria: (1) a log_2_-transformed fold change greater than 0.585, and (2) an FDR value below 0.05. GO analysis was performed using the *Metascape* web tool with default settings.

### Processing of WGBS data from placentas and identification of DMPs

For WGBS DNA methylation sequencing data, we used the same pipeline as for PBAT data, and 3× coverage was used as the read-depth cutoff for downstream analysis.

To identify DMPs between the IS and NI groups, only promoters with at least 3 CpG sites were considered. DMPs were identified using the following criteria: (1) an average methylation difference exceeding 10%, and (2) a two-tailed Student’s *t*-test *P*-value ≤ 0.05. Notably, for a given candidate region, all CpG sites in all samples from a given group were used to calculate the average methylation level and perform statistical tests.

### Processing of scRNA-seq data from neonatal UCB cells

The raw scRNA-seq data were analyzed using the *count* command in the 10× Genomics software *Cell Ranger* (version 8.0.0). The sequencing reads were mapped to the human reference genome (GRCh38).

Upon acquiring the processed scRNA-seq data, we used *Seurat* to isolate non-empty droplets, ensuring that the detected genes numbered over 200 and were expressed in at least three cells. The cells were then filtered using three criteria: (1) the number of detected genes exceeded 200 but was below 10,000, (2) the number of detected UMIs exceeded 1000 but was below 75,000, and (3) mitochondrial transcripts constituted < 10% of the total. To eliminate potential doublets, we used the Python package *scrublet*^95^, identifying doublets with an expected rate of 6% per library and excluding cells with a *doubletScore* above the 90th percentile. After these steps, we retained high-quality single cells that exhibited more than 500 detected genes but fewer than 60,000 UMIs for further analysis.

Gene expression levels were quantified using UMI counts per million mapped reads (CPM), calculated by dividing the UMI count of a specific gene in a single cell by the total UMI count in that cell, then multiplying by 100,000. For this study, the gene expression levels were transformed as log_2_(CPM + 1).

### Identification of cell types and cell type-specific marker genes among neonatal UCB cells

The scRNA-seq data, quantified as raw UMI counts, were imported into *Seurat* to identify cell types in neonatal UCB cells. UMI count data were normalized and scaled using the *NormalizeData* and *ScaleData* functions in *Seurat* with default parameters. HVGs were identified using the *FindVariableFeatures* function, and PCA was then performed using the *RunPCA* function with the selected HVGs. To minimize the influence of individual heterogeneity on cell type identification, the Harmony algorithm was implemented using the *IntegrateLayers* function with the ‘*HarmonyIntegration*’ method^96^. The Harmony algorithm converged after three iterations. The first 20 Harmony dimensions were used for non-linear dimensionality reduction analysis via UMAP, performed using the function *RunUMAP*. Unsupervised clustering analysis was performed to separate all single cells into 26 (for all UCB cells) or 38 (for HSPCs) distinct clusters using the function *FindClusters* with the clustering ‘*resolution*’ parameter set to 1 (for all UCB cells) or 2 (for HSPCs).

Cell type-specific marker genes were identified by analyzing DEGs across different cell types using the function *FindAllMarkers* with the Wilcoxon rank sum test. Genes were classified as DEGs if they met the following criteria: (1) a log_2_-transformed fold change greater than 1, (2) an FDR below 0.05, and (3) expression in > 25% of cells in the corresponding cell type. For visualization, the mean expression level of each gene in a specific cell type was calculated, and expression values were scaled to a range of 0–1 across all cell types.

### Identification of DEGs in neonatal UCB cells between the IS and NI groups

To identify DEGs in neonatal UCB cells between the two groups, log_2_-transformed CPM data were analyzed using the Wilcoxon rank sum test implemented with the *FindAllMarkers* function. Genes were classified as DEGs if they satisfied three criteria: (1) a log_2_-transformed fold change exceeding 0.25, (2) an FDR below 0.05, and (3) detectable expression in at least 25% of cells in the corresponding group. GO analysis of these DEGs was performed using the *Metascape* web tool with default parameters^97^.

### Calculation of gene module scores

This study involved two public gene sets: (1) hallmark genes related to TNF-α signaling via NFκB obtained from the GSEA database and (2) hematopoiesis-related TF genes, including *SOX4*, *MEF2C*, *HIVEP3*, *TCF7L2*, *FOSB*, *JUND*, *HES1*, *IRF1*, *TCF4*, *ETS1*, *CSRNP1*, *TSC22D3*, *TFEC*, and *EGR1*, which were highly expressed in LMPPs compared to GMPs, and affiliated with the myeloid lineage. These TFs were collected from a previous study^52^. We evaluated the gene module score of a given gene set in individual cells using the *AddModuleScore* function in *Seurat*.

## Supplementary information

Supplementary Table S1

Supplementary Table S2

Supplementary Table S3

Supplementary Table S4

Supplementary Table S5

Supplementary Figures

## Data Availability

The raw sequencing data reported in this study have been deposited in the Genome Sequence Archive (GSA)-Human under accession number HRA010475. The processed scRNA-seq, PBAT, bulk RNA-seq, and WGBS data reported in this study have been deposited in the Gene Expression Omnibus under accession number GSE289648.

## Code availability

Publicly available software used in this study are listed in the “Materials and methods” section.
