# Supplementary material for: Maternal acute SARS-CoV-2 infection impairs preimplantation embryo development and reprograms the early offspring hematopoietic system
Source: Cell Discov. 2025 Dec 23;11:104. doi: 10.1038/s41421-025-00856-3 (PMC12728194; doi:10.1038/s41421-025-00856-3)
Supplement: Supplementary file 6 — Supplementary Figures [file 41421_2025_856_MOESM6_ESM.pdf]

a

Embryo	Embryo day	Woman age	UCB donor ID
NI_Embryo6	Day 6	<35	/
NI_Embryo7	Day 5	26	/
NI_Embryo8	Day 5	37	/
NI_Embryo9	Day 6	29	/
NI_Embryo10	Day 6	30	/
IS_Embryo1	Day 6	40	/
IS_Embryo2	Day 5	28	IS.UCB_Donor3
IS_Embryo3	Day 6	32	IS.UCB_Donor4

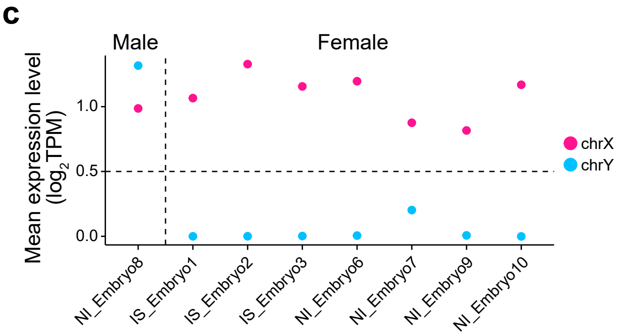

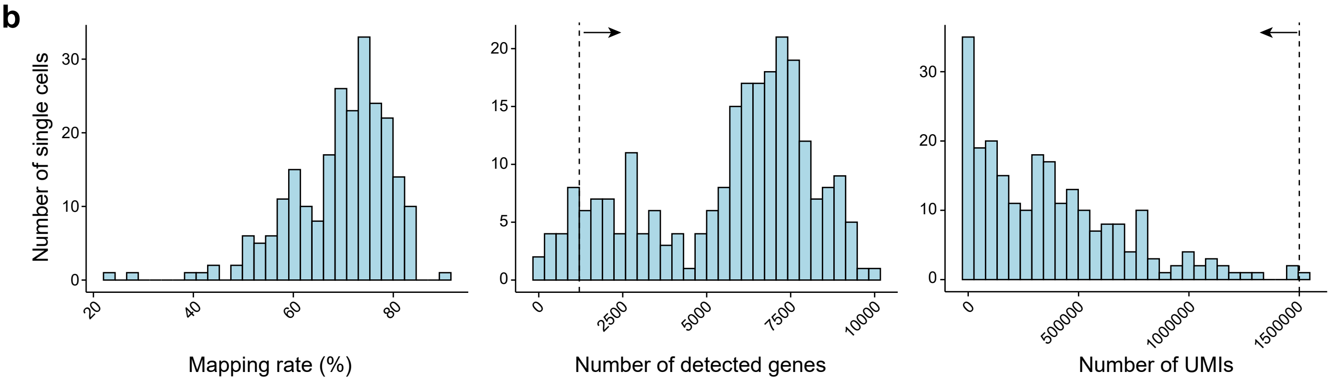

Figure S1. Basic information of samples and donors, and quality control information of scRNA-seq data of blastocysts.
(a) Table showing the basic information of samples and donors.
(b) Density plots showing the mapping rate (left), the number of detected genes (middle), and the number of UMIs (right) among all single cells before filtering. Dashed lines indicate the standard to filter high-quality single cells. These cells are collected from blastocysts.
(c) Dot plot showing the average expression level of genes in X and Y chromosomes.

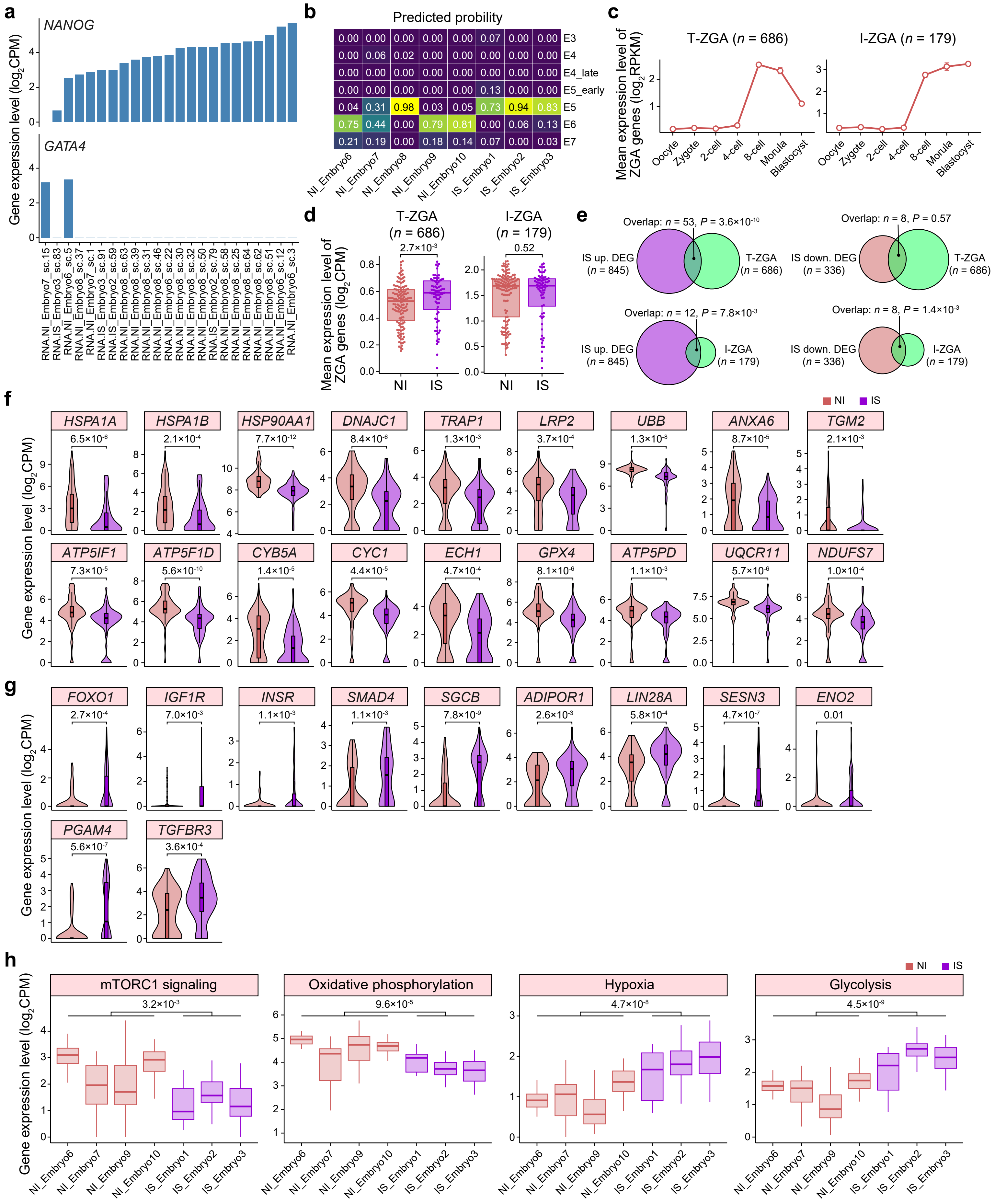

a
NANOG
GATA4
Gene expression level (log2CPM)
b
Predicted probility
E3
E4
E4_late
E5_early
E5
E6
E7
NI_Embryo6
NI_Embryo7
NI_Embryo8
NI_Embryo9
NI_Embryo10
IS_Embryo1
IS_Embryo2
IS_Embryo3
c
T-ZGA (n = 686)
I-ZGA (n = 179)
Mean expression level of ZGA genes (log2RPKM)
Oocyte
Zygote
2-cell
4-cell
8-cell
Morula
Blastocyst
d
T-ZGA (n = 686)
I-ZGA (n = 179)
2.7×10-3
0.52
Mean expression level of ZGA genes (log2CPM)
NI
IS
e
Overlap: n = 53, P = 3.6×10-10
IS up. DEG (n = 845)
T-ZGA (n = 686)
Overlap: n = 8, P = 0.57
IS down. DEG (n = 336)
Overlap: n = 12, P = 7.8×10-3
I-ZGA (n = 179)
Overlap: n = 8, P = 1.4×10-3
f
NI
IS
HSPA1A
HSPA1B
HSP90AA1
DNAJC1
TRAP1
LRP2
UBB
ANXA6
TGM2
ATP5IF1
ATP5F1D
CYB5A
CYC1
ECH1
GPX4
ATP5PD
UQCR11
NDUFS7
Gene expression level (log2CPM)
g
FOXO1
IGF1R
INSR
SMAD4
SGCB
ADIPOR1
LIN28A
SESN3
ENO2
PGAM4
TGFBR3
h
mTORC1 signaling
Oxidative phosphorylation
Hypoxia
Glycolysis
3.2×10-3
9.6×10-5
4.7×10-8
4.5×10-9

Figure S2. scRNA-seq data of blastocysts revealing the molecular evidence of developmental delay in good-quality blastocytes from the IS group.
(a) Barplots showing the expression level of *NANOG* (EPI marker gene) and *GATA4* (PE marker gene).
(b) Heatmap showing the probability of single cells being assigned to specific developmental days, as calculated by the established machine-learning model, corresponding to fig. 2e and 2f.
(c) Line plots showing the average expression level of ZGA-related genes (T-ZGA and I-ZGA). The expression data is collected from the previous study. The number of T-ZGA- or I-ZGA-related genes are indicated in brackets. Data are shown as mean ± SEM.
(d) Boxplots showing the expression level of ZGA-related genes (T-ZGA and I-ZGA) in TE cells (right, n = 201) from NI and IS groups. One-tailed Wilcoxon rank sum test P value is indicated.
(e) Venn plots showing the overlap between ZGA-related genes (T-ZGA and I-ZGA) and DEGs. These DEGs are identified in blastocysts between IS and NI groups.
(f and g) Violin plots showing the expression level of representative downregulated (f) and upregulated (g) DEGs in blastocysts between IS and NI groups. Two-tailed Student's t-test P values are indicated.
(h) Boxplots showing the gene expression level of key pathways in each embryo from IS and NI groups. After removing the confounding factor of embryo sources with the linear regression method, two-tailed Student's t-test P values are indicated.

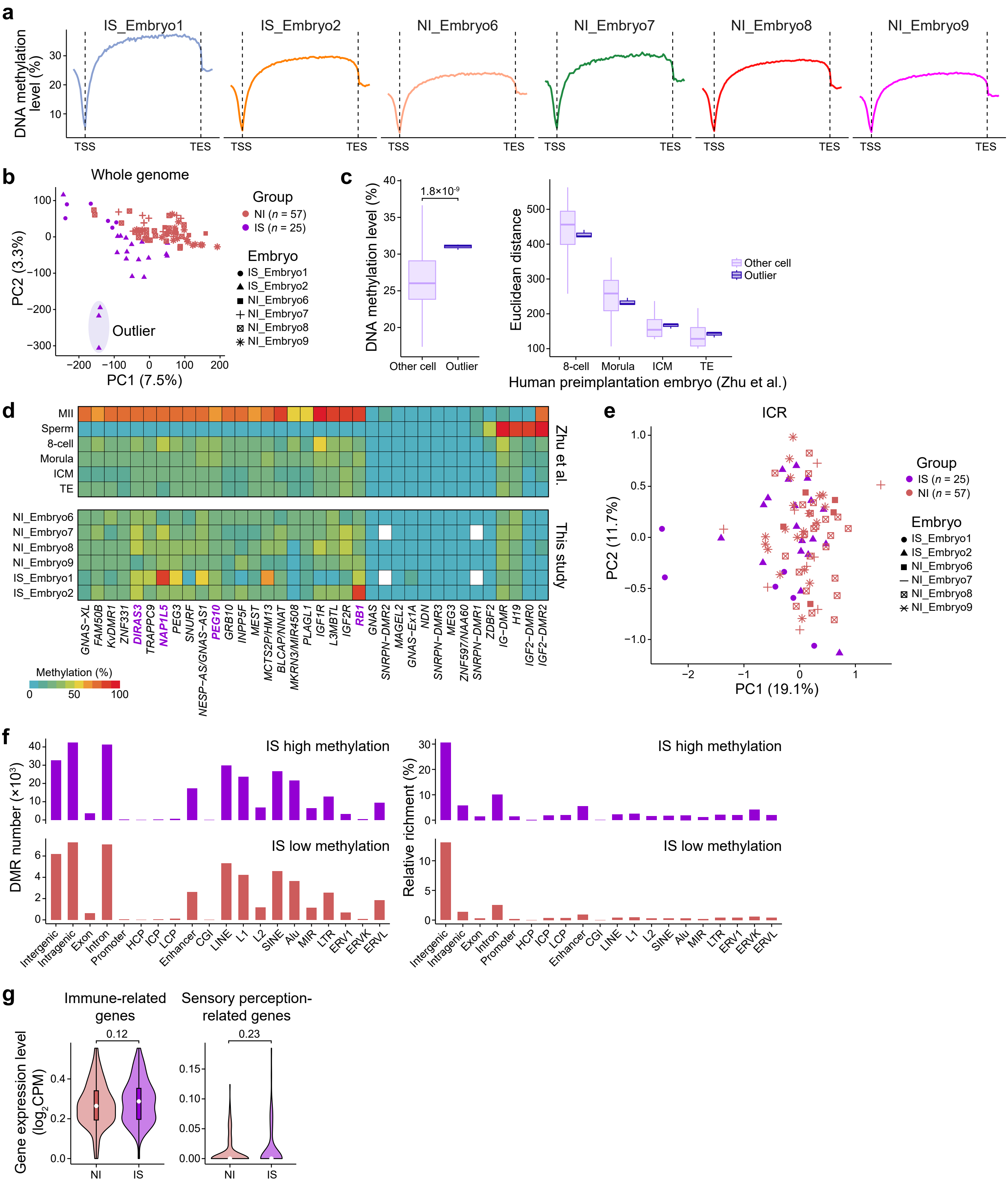

a
IS_Embryo1
IS_Embryo2
NI_Embryo6
NI_Embryo7
NI_Embryo8
NI_Embryo9
DNA methylation level (%)
TSS
TES
b
Whole genome
PC1 (7.5%)
PC2 (3.3%)
Outlier
Group
NI (n = 57)
IS (n = 25)
Embryo
IS_Embryo1
IS_Embryo2
NI_Embryo6
NI_Embryo7
NI_Embryo8
NI_Embryo9
c
DNA methylation level (%)
1.8×10-9
Other cell
Outlier
Euclidean distance
8-cell
Morula
ICM
TE
Human preimplantation embryo (Zhu et al.)
d
MII
Sperm
8-cell
Morula
ICM
TE
Zhu et al.
NI_Embryo6
NI_Embryo7
NI_Embryo8
NI_Embryo9
IS_Embryo1
IS_Embryo2
This study
GNAS-XL
FAM50B
KvDMR1
ZNF331
DIRAS3
TRAPPC9
NAP1L5
PEG3
SNURF
NESP-AS/GNAS-AS1
PEG10
GRB10
INPP5F
MEST
MCTS2P/HM13
BLCAP/NNAT
MKRN3/MIR4508
PLAGL1
IGF1R
L3MBTL
IGF2R
RB1
GNAS
SNRPN-DMR2
MAGEL2
GNAS-Ex1A
NDN
SNRPN-DMR3
MEG3
ZNF597/NAA60
SNRPN-DMR1
ZDBF2
IG-DMR
H19
IGF2-DMR0
IGF2-DMR2
Methylation (%)
e
ICR
PC1 (19.1%)
PC2 (11.7%)
Group
IS (n = 25)
NI (n = 57)
Embryo
IS_Embryo1
IS_Embryo2
NI_Embryo6
NI_Embryo7
NI_Embryo8
NI_Embryo9
f
IS high methylation
IS low methylation
DMR number (×10³)
Relative richment (%)
Intergenic
Intragenic
Exon
Intron
Promoter
HCP
ICP
LCP
Enhancer
CGI
LINE
L1
L2
SINE
Alu
MIR
LTR
ERV1
ERVK
ERVL
g
Immune-related genes
Sensory perception-related genes
Gene expression level (log2CPM)
0.12
0.23
NI
IS

Figure S3. Single-cell DNA methylation characteristics of blastocysts.
(a) Line plots showing the average DNA methylation level in each embryo.
(b) PCA plot showing the cell distribution based on the DNA methylation level in the whole genome. The outlier blastocysts from the IS group are highlighted. The number of single cells is indicated in the bracket.
(c) Left, boxplot showing the average DNA methylation level of the whole genome in outlier and other blastocysts from the IS group. Two-tailed Student's *t*-test *P* value is indicated. Right, boxplot showing the Euclidean distance within the PCA space between outlier and other blastocysts from the IS group and publicly available human preimplantation embryos. Two-tailed Student's *t*-test *P* values are calculated ($P > 0.05$ for all comparisons between outlier and other IS blastocysts).
(d) Top, heatmap showing the single-cell DNA methylation of ICRs in human germ cells and preimplantation embryos collected from the previous study. Bottom, heatmap showing the single-cell DNA methylation of ICRs in blastocysts from IS and NI groups. The ICRs with the significant (two-tailed Student's *t*-test) different methylation level greater than 10% are colored with purple.
(e) PCA plot showing the cell distribution based on the DNA methylation level of ICRs.
(f) Barplots showing the number (left) and relative enrichment (right) of DMRs.
(g) Violin plots showing the average expression level of immune- (left) and sensory perception-related genes (right) in blastocysts from IS and NI groups. Two-tailed Student's *t*-test *P* values are indicated.

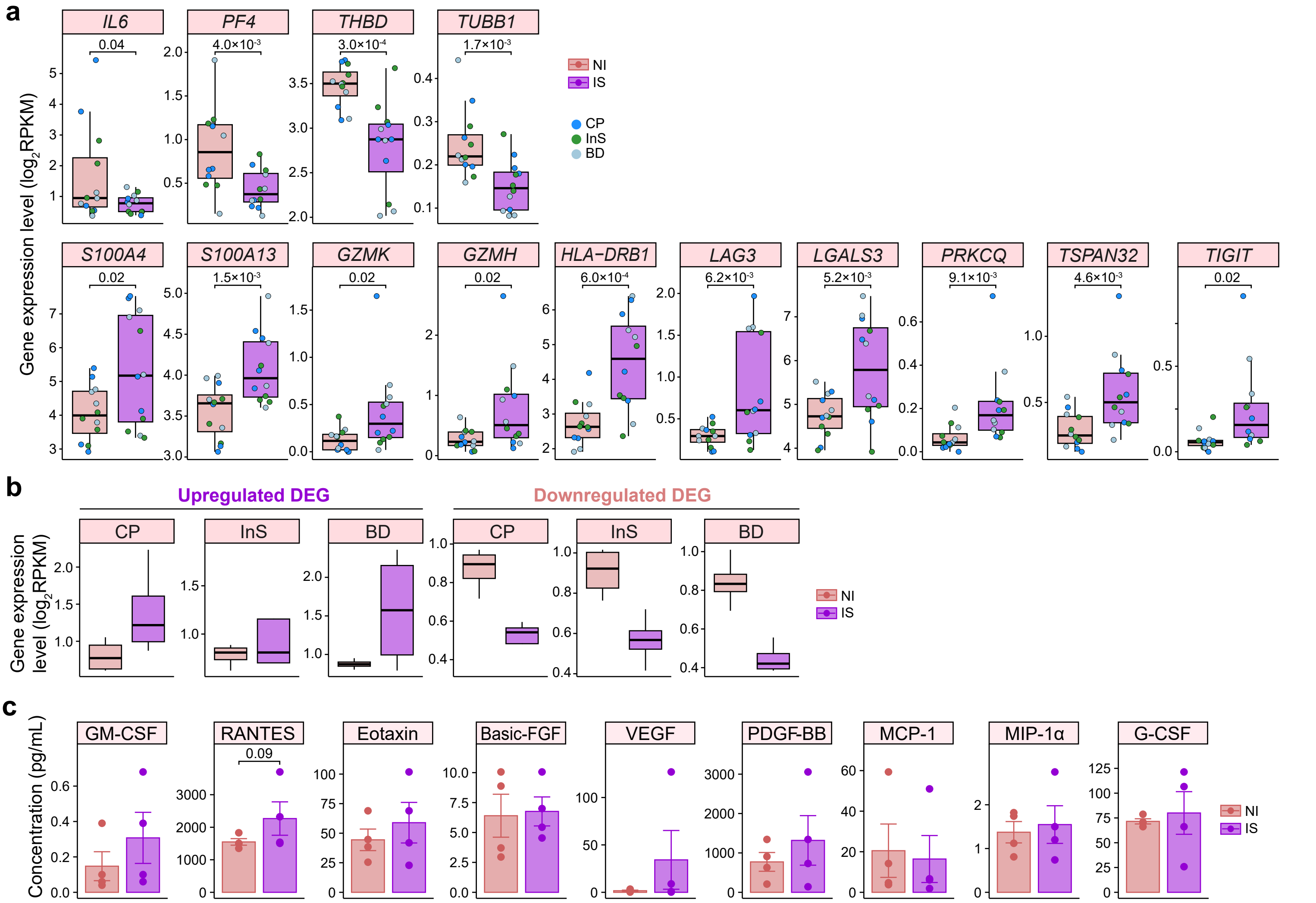

Figure S4. The IS placentas displaying the immune response activation.

(a) Boxplots showing the expression level of representative DEGs between IS and NI placentas. One-tailed Student's *t*-test *P* values are indicated.

(b) Boxplots showing the average expression level of upregulated (left) and downregulated (right) DEGs between IS and NI groups in distinct anatomical structures of placentas.

(c) Barplots showing the concentration of representative cytokines in UCB serum. Each dot indicates each neonate. Data are shown as mean ± SEM. One-tailed Wilcoxon rank sum test *P* values are indicated.

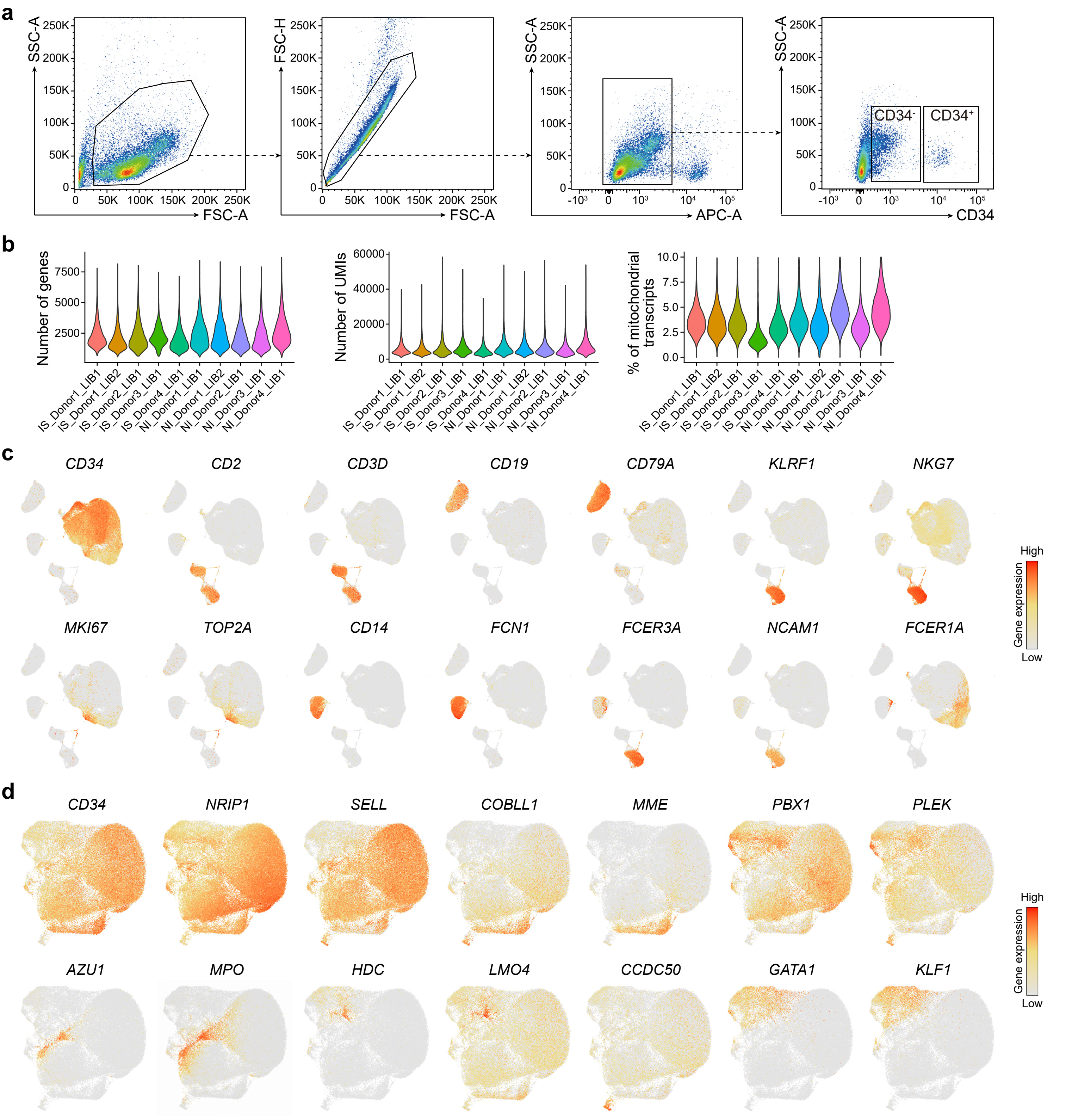

Figure S5. The single-cell transcriptome characteristics of UCB cells.
(a) Scatter plots showing the gating strategy to obtain immune cells and HSPCs.
(b) Violin plots showing the quality control information of scRNA-seq data of UCB cells. These single cells are displayed after filtering.
(c and d) UMAP plots showing the expression level of representative well-known marker genes among immune cells (c) and HSPCs (d).

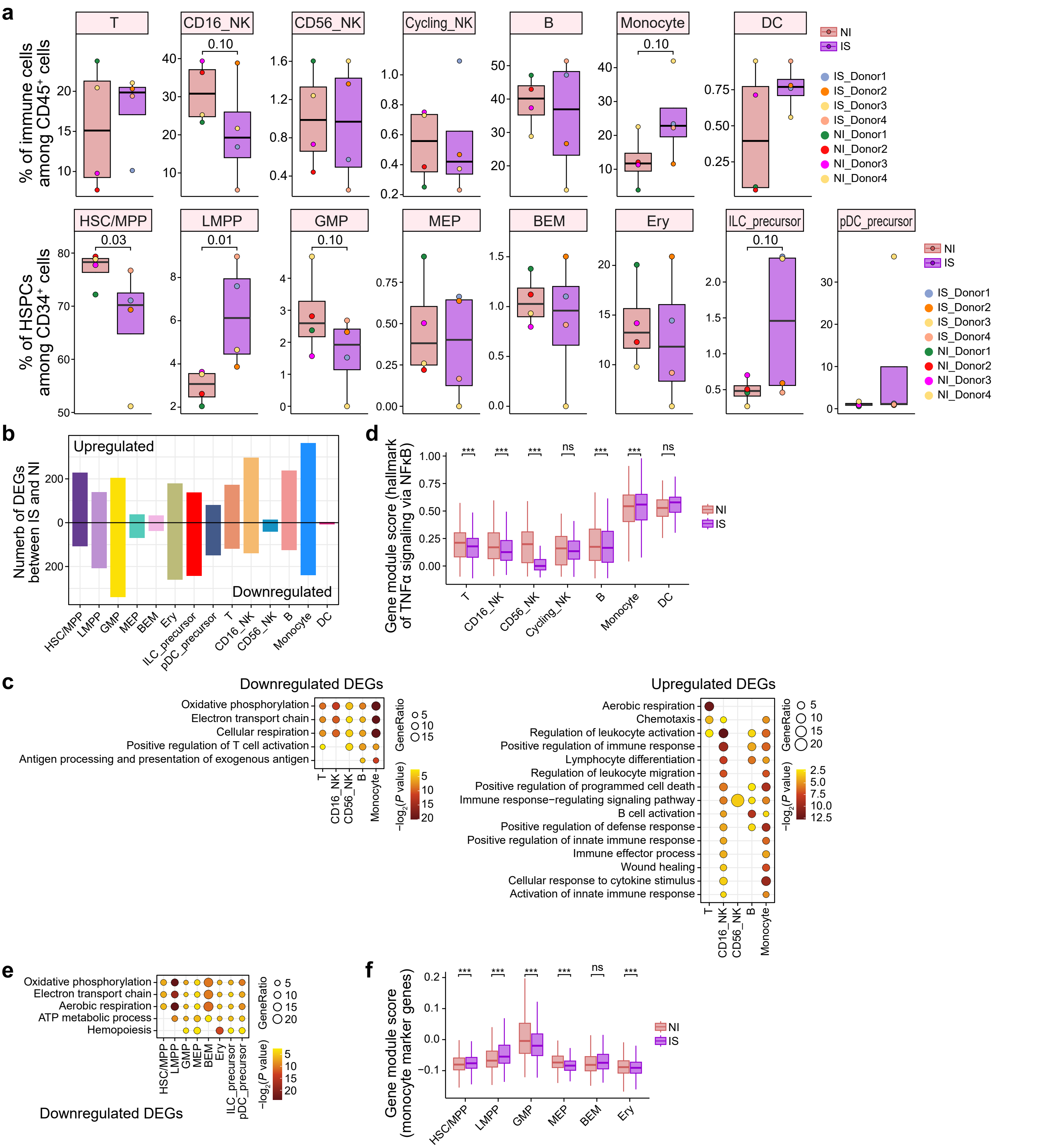

Figure S6. scRNA-seq data of UCB cells revealing transcriptomic differences between IS and NI groups.
(a) Boxplots showing the percentage of distinct cell types among immune cells (top) and HSPCs (bottom).
(b) Barplot showing the number of DEGs between IS and NI groups in distinct UCB cell types.
(c) Dot plots showing GO terms (biological process) corresponding to downregulated (left) and upregulated (right) DEGs in immune cells from the IS group compared to the NI group. Dot size indicates the ratio of related genes among inputs and dot color indicates the statistical significance.
(d) Boxplot showing the gene module score of the hallmark of TNF-α signaling via NFκB among immune cells. Two-tailed Student's *t*-test *P* values are indicated.
(e) Dot plot showing GO terms (biological process) corresponding to downregulated DEGs in HSPCs from the IS group compared to the NI group. Dot size indicates the ratio of related genes among inputs and dot color indicates the statistical significance.
(f) Boxplot showing the gene module score of monocyte-specific marker genes. These marker genes are identified by this study. Two-tailed Student's *t*-test *P* values are indicated.

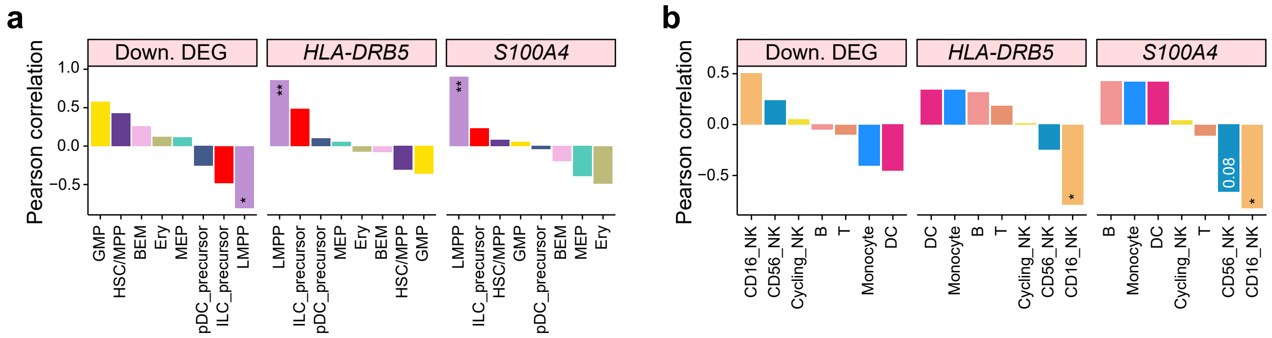

Figure S7. The correlation between the pro-inflammatory response within placentas and the abundance of distinct CD34⁺/CD45⁺ cell types among UCB cells.

(a) Bar plots showing the Pearson's correlation coefficient between the abundance of distinct CD34⁺ cell types and the expression of (all or representative) upregulated/downregulated DEGs within IS placentas. Pearson's correlation test significance is calculated, in which $*P < 0.05$, $**P < 0.01$, and $***P < 0.001$.

(b) Bar plots showing the Pearson's correlation coefficient between the abundance of distinct CD45⁺ cell types and the expression of (all or representative) upregulated/downregulated DEGs within IS placentas. Pearson's correlation test significance is calculated, in which $*P < 0.05$.
